# Development of Novel Galactosylated PLGA Nanoparticles for Hepatocyte Targeting Using Molecular Modelling

**DOI:** 10.3390/polym12010094

**Published:** 2020-01-04

**Authors:** Cláudia D. Raposo, Rita Costa, Krasimira T. Petrova, Catarina Brito, Marcus T. Scotti, M. Margarida Cardoso

**Affiliations:** 1 LAQV-REQUIMTE, Departamento de Química, Faculdade de Ciências e Tecnologia, Universidade NOVA de Lisboa, Quinta da Torre, 2829-516 Caparica, Portugal; 2 IBET, Instituto de Biologia Experimental e Tecnológica, Apartado 12, 2781-901 Oeiras, Portugal; 3 Instituto de Tecnologia Química e Biológica António Xavier, Universidade Nova de Lisboa, Av. da República, 2780-157 Oeiras, Portugal; 4 Departamento de Química, Centro de Ciências Exatas e da Natureza, Universidade Federal da Paraíba Campus I, João Pessoa-PB 58051-900, Brazil

**Keywords:** hepatocyte targeting nanoparticles, doxorubicin delivery, galactosylated nanoparticles, surface modified PLGA nanoparticles, molecular modelling for drug targeting

## Abstract

Doxorubicin-loaded PLGA nanoparticles conjugated with a new galactose-based ligand for the specific recognition by human hepatoma cellular carcinoma cells (Hep G2) were successfully produced. The new targeting compound was selected using molecular docking combined with quantum chemical calculations for modelling and comparing molecular interactions among the H1 subunit of the asialoglycoprotein receptor containing the carbohydrate recognition domain and the ligand. The ligand, bis(1-*O*-ethyl-β-D-galactopyranosyl)amine, was synthetized, characterized, and subsequently linked to PLGA. Unloaded (PLGA-di-GAL NP) and doxorubicin-loaded (DOX-PLGA-di-GAL NP) nanoparticles were prepared using an emulsion method and characterized. The produced DOX-PLGA-di-GAL NP are spherical in shape with a size of 258 ± 47 nm, a zeta potential of −62.3 mV, and a drug encapsulation efficiency of 83%. The in vitro drug release results obtained show a three-phase release profile. In vitro cell studies confirmed the interaction between Hep G2 cells and PLGA-di-GAL NP. Cell cytotoxicity tests showed that unloaded NP are nontoxic and that DOX-PLGA-di-GAL NP caused a decrease of around 80% in cellular viability. The strategy used in this work to design new targeting compounds represents a promising tool to develop effective hepatocyte targeting drug delivery systems and can be applied to other tissues/organs.

## 1. Introduction

Delivering drugs is a major challenge in clinical development since drugs are frequently limited by dose-limiting toxicity and to a low specificity to the desirable organ or tissue. Nanoparticles (NP) can be used as drug carriers since they can circulate in the blood or cross cell membranes and be internalized. Designing NP containing moieties capable to be recognized by proteins that are expressed in target cells is a viable synthesis strategy to obtain specificity and efficiency. Apart from improving the drug therapeutic index, drug targeting by nanoparticles can offer all the advantages of drug carrier systems: the incorporation of several drugs for combination therapy or different therapeutic modalities, a controlled release of drug, the ability to carry hydrophobic drugs, and a protection against drug degradation, while causing lower systemic toxicity since drugs are biologically unavailable during transit in systemic circulation [1,2,3,4]. Liver diseases are particularly difficult to treat as efficient drugs are not currently available [5]. Liver cancer, for instance, is the fifth most common cancer in the Unites States with increasingly mortality rates and with a five-year survival rate of 18% [6]. Safer and more efficient liver therapeutic agents are urgently needed to treat these malignancies in order to allow a significant increase in life expectancy. Hepatocytes are parenchymal cells in the liver that have an abundant receptor, named asialoglycoprotein receptor (ASGP-R), responsible for recognition and internalization of glycoproteins containing terminal galactose or *N*-acetylgalactosamine residues [7]. Targeting galactose- or *N*-acetylgalactosamine-derived therapeutic agents to ASGP-R can provide safer and more effective liver therapies [8,9,10,11,12,13]. 

In this study we focused on the synthesis of a new compound containing two units of galactose that is covalently attached to poly(DL-lactide-co-glycoglide), PLGA, a biodegradable and biocompatible polymer [14], to produce drug-loaded NP for hepatocyte targeting. First, we evaluate the recognition of the purposed compound using molecular modelling comparing with other molecules known to be recognized by ASGP-R. Then, we synthesized the new compound and the polymeric conjugate and prepared the polymeric NP loaded with doxorubicin (DOX), an anthracycline commonly used in the treatment of a wide range of cancers, including many types of carcinomas (solid tumors). It works by inhibiting nucleic acids synthesis within cancer cells but it is rapidly cleared from the blood and leads to various dose-limiting side effects [15,16]. After NP characterization, the produced NP were further used for in vitro drug release studies and in vitro assays in mammalian cell line cultures to access their capability to be recognized by human hepatoma cells (Hep G2), a suitable in vitro model to study the interactions of galactose with ASGPR, and their effect on cell viability.

## 2. Materials and Methods

### 2.1. Molecular Modelling

**Figure 1:**
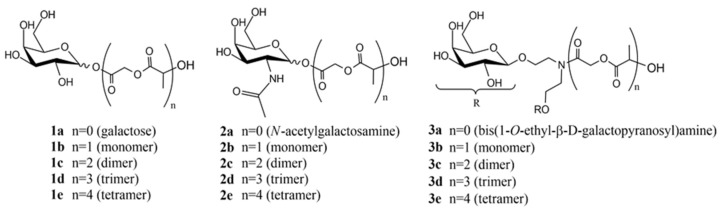
Ligands chemical structures used for the docking studies.

In order to predict the recognition of the ligands by the asialoglycoprotein receptor, docking of the ligands on the carbohydrate recognition domain of the H1 subunit of the ASGP-R were performed [17]. The ligands′ 3D structures were energy-minimized using Gamess 12.0 (Cambridge Soft interface, Perkin Elmer, Cambridge, MA, USA) by employing semi-empirical method AM1. Then, a conformational analysis of each ligand was performed in order to select its global minimum, using MMFF method of Spartan 1.2.0. Docking studies were performed using AutoDock Vina (Scripps Research, La Jolla, CA, USA), an automated docking program designed for the prediction of the binding among small molecules and the receptor with a known 3D structure. Auto Dock Tools 4.0 was used to prepare the ligands and the macromolecule. The macromolecule used for the studies was 1DV8 from Protein Data Bank, and all the water molecules were removed. Gasteiger charges and polar hydrogen (noBondOrder method) were added to the ligands and non-polar hydrogens were merged. Three ligands were tested: one with a galactose unit (Figure 1(**1a**–**e**)) and one with an *N*-acetylgalactosamine unit (Figure 1(**2a**–**e**)), as controls, since these residues are known to be recognized by the ASGPR [18], and our proposed ligand, bis(1-*O*-ethyl-β-D-galactopyranosyl)amine, **3a**, containing two galactose units, (Figure 1(**3a**–**e**)).

### 2.2. Materials

Dichloromethane (DCM) was distilled from CaH_2_ and pyridine from KOH prior to use [19]. PLGA (50:50, *M*_w_ 40,000–70,000), DOX, poly(vinyl alcohol) (PVA, *M*_w_ 30,000–70,000) and galactose were from Sigma-Aldrich (Saint Louis, MO, USA). Rhodamine B of 95% purity was obtained from Fluka (Buchs, Switzerland). All other materials or solvents used were of analytical grade. Column chromatography was performed on silica gel 60 Å (Carlo Erba, particle size 40–63 μm, Val de Reuil, France). Melting points were determined using an Electrothermal Melting Point Apparatus (Cole-Parmer, Staffordshire, UK). Optical rotations were measured using an AA-1000 Polarimeter Optical Activity LTD (Cambridgeshire, UK) at 589 nm (0.5 dm cell). The concentrations (*c*) are expressed in g/102 mL. FTIR spectra were recorded on a 1000 FT-IRBX Perkin-Elmer Spectrum (Villepinte, France) apparatus for solid and liquid compounds in KBr dispersions or NaCl windows, respectively. NMR spectra were recorded on a Bruker ARX 400 spectrometer (400 MHz for 1H NMR and 101 MHz for 13C NMR), (Coventry, UK). Chemical shift values (δ) are expressed in parts per million (ppm) and reported downfield from TMS (0.00 ppm) for CDCl3 or the residual peak of DMSO (2.50 ppm) for DMSO-d6. Exact mass spectra, ESI-FIA-TOF MS (EM), were recorder on a Bruker Microtof, using electrospray ionization, flow injection analysis, and time-of-flight detector. Mass spectra MALDI-TOF MS (matrix-assisted laser desorption ionization–time of flight–mass spectrometry) were recorded on a Voyager-DETM PRO Biospectrometry Workstation (Foster City, CA, USA), using a DHB matrix and reflector positive ion mode. All cell culture reagents were from Thermo Fisher Scientific, as well as the PrestoBlue™ Viability Reagent reduction assay. The cell lines HepG2/C3A and HEK293T were acquired from ATCC (Manassas, VA, USA).

### 2.3. Ligand and Polymer Synthesis

**Scheme 1:**
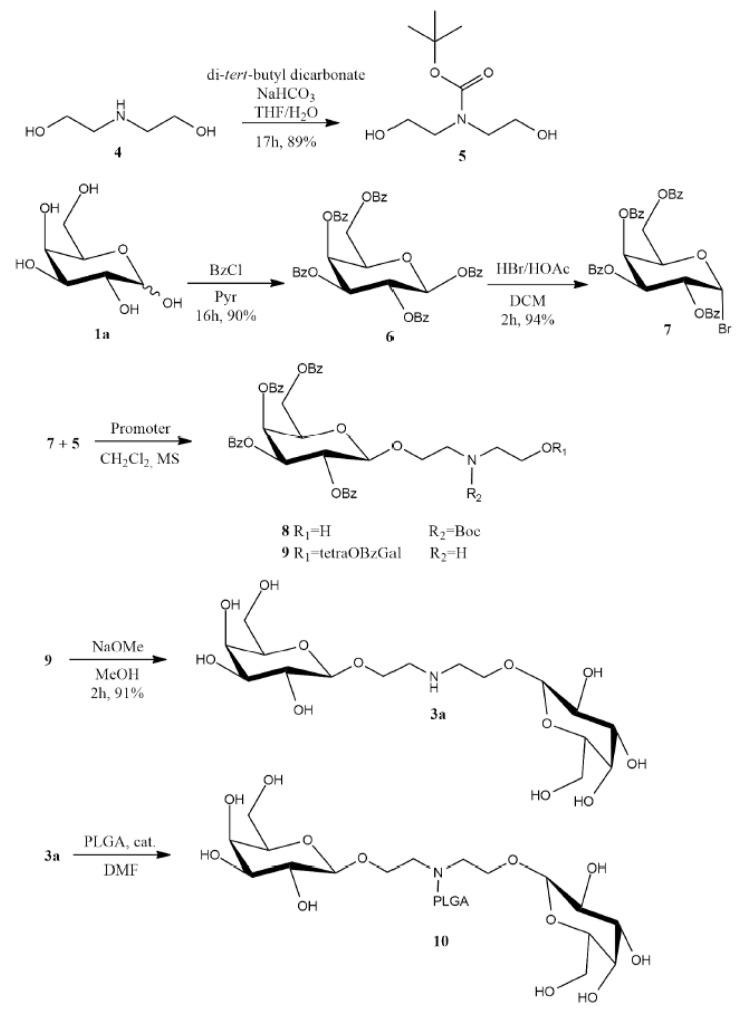
Synthetic strategy to prepare ligand **3a** and **PLGA-di-GAL**, **10**. Promoter = Ag_2_CO_3_ or Ag_2_SO_4_; catalyst = montmorillonite K10 or methanesulfonic acid.

In order to produce bis(1-*O*-ethyl-β-D-galactopyranosyl)amine-PLGA conjugate (PLGA-di-GAL), **10**, the ligand bis(1-*O*-ethyl-β-D-galactopyranosyl)amine, **3a**, was synthetized from diethanolamine and galactose, and subsequently linked to PLGA using the synthetic strategy presented in Scheme 1 and described below.

Synthesis of *N*-Boc-diethanolamine, (tert-butyl bis(2-hydroxyethyl)carbamate), **5**.

Initially, diethanolamine, **4**, is protected with tert-butyloxycarbonyl group (Boc), using the procedure described in the literature [20], affording *N*-Boc-diethanolamine, **5**. To a solution of diethanolamine (4, 0.4 g, 3.8 mmol) in water/THF (1:1, 5 mL), sodium bicarbonate (0.98 g, 11.7 mmol) was added. The mixture was stirred for 25 min and di-tert-butyldicarbonate (1.0 g, 4.6 mmol) was added. Upon completion (17 h), the reaction mixture was filtered, concentrated and purified by flash column chromatography (100:100:1, AcOEt/acetone/water) to give **5** as a colorless oil (0.70 g, 89%), identical in all respects to those described in literature. 

^1^H NMR (400 MHz, D_2_O) δ 3.70 (s, 4H, 2xHO–CH_2_), 3.41 (s, 4H, 2xN–CH_2_), 1.45 (s, 9H, 3xCH_3_) (Figure S2); ^13^C NMR (100 MHz, D_2_O) δ 157.6 (C=O), 81.6 (C_q_), 59.6(CH_2_–OH), 59.4 (CH_2_–OH), 49.9 (CH_2_–N), 49.5 (CH_2_–N), 27.7 (3xCH_3_) (Figure S3).

Synthesis of 1,2,3,4,6-penta-*O*-benzoyl-α-D-galactopyranose, **6.**

D-galactose (1, 0.55 g, 3.04 mmol) was dissolved in pyridine (7.5 mL) under vigorous stirring and argon atmosphere. The reaction was cooled in an ice bath and benzoyl chloride (2.1 mL, 18.2 mmol) was added dropwise. The reaction mixture was allowed to warm to room temperature. Upon completion (16 h), the reaction was diluted with DCM (25 mL), washed three times with HCl 1 M, and once with saturated solution of sodium bicarbonate. The organic layer was dried over anhydrous sodium sulphate, concentrated and purified by flash column chromatography (5:1, hexane/AcOEt) to afford **6** as a white foam (1.76 g, 90%), identical in all respects to those described in the literature.

^1^H NMR (400 MHz, CDCl_3_) δ 8.23–7.14 (m, 25H, Ar), 6.96 (d, *J* = 3,4 Hz, 1H, H-1), 6.19 (s, 1H, H-4), 6.13 (dd, *J* = 10,7, 3,0 Hz, 1H, H-3), 6.03 (dd, *J* = 10,7, 3,5 Hz, 1H, H-2), 4.84 (t, *J* = 6,5 Hz, 1H, H-5), 4.64 (m, 1H, H-6A), 4.43 (m, 1H, H-6_B_) (Figure S4); ^13^C NMR (101 MHz, CDCl_3_) δ 165.9, 165.7, 165.6, 165.5, 164.6 (CO), 133.9, 133.8, 133.5, 133.4, 133.3, 130.2, 130.0, 129.9, 129.8, 129.3, 129.0, 129.0, 128.9, 128.8, 128.8, 128.7, 128.4, 128.4 (C-Ar), 90.7 (C-1), 69.5 (C-5), 68.6, 68.5 (C-4, C-3), 67.7 (C-2), 61.9 (C-6) (Figure S5); DEPT, COSY, HMQC and HMBC (CDCl_3_) spectra presented in Figures S6–S9.

Synthesis of 2,3,4,6-tetra-*O*-benzoyl-α-D-galactopiranosyl bromide, **7**.

Penta-O-benzoylgalactopyranose (6, 0.60 g, 0.86 mmol) was dissolved in DCM (9 mL) in an argon atmosphere. The reaction was then cooled in an ice bath and hydrobromic acid solution (33% in acetic acid, 1.51 mL, 8.60 mmol) was added dropwise. The reaction was allowed to warm to room temperature and upon completion (2 h) was diluted with dichloromethane (10 mL) and ice was added. The organic layer was softly washed three times with saturated solution of sodium bicarbonate, dried over anhydrous sodium sulfate, and concentrated to dryness, giving **7** as a white foam (0.54 g, 94% without purification), identical in all respects to those described in the literature. 

Rf= 0.55 (3:1 hexane/AcOEt); FTIR (KBr, cm^−1^), υ: 3064, 2962, 2925 (C–H st), 1733, 1727 (C=O), 1601 (Ar), 1316, 1267 (CO–O as st, O–C–C as st), 1105, 1094 (C–O–C as st), 1068 (C–O–C sym st) (Figure S10); m.p. 69–71 °C; ${\left[\alpha \right]}_{D}^{27\;{}^{\circ}C}$ = +168.2 (c 1, CHCl_3_); ^1^H NMR (400 MHz, CDCl_3_) δ 8.17–7.16 (m, 20H, Ar), 6.98 (d, *J* = 3.8 Hz, 1H, H-1), 6.12 (s, 1H, H-4), 6.06 (dd, *J* = 10.4, 3.0 Hz, 1H, H-3), 5.67 (dd, *J* = 10.4, 3.8 Hz, 1H, H-2), 4.92 (t, *J* = 6.2 Hz, 1H, H-5), 4.64 (dd, *J* = 11.5, 6.8 Hz, 1H, H-6_A_), 4.47 (dd, *J* = 11.5, 6.0 Hz, 1H, H-6B) (Figure S11); ^13^C NMR (101 MHz, CDCl_3_) δ 165.9, 165.6, 165.4, 165.3 (C=O), 133.8, 133.4, 133.3, 130.0, 129.9, 129.8, 129.7, 129.3, 128.8, 128.7, 128.6, 128.5, 128.3 (C-Ar), 88.3 (C-1), 71.8 (C-5), 68.9 (C-3), 68.6 (C-2), 68.1 (C-4), 61.7 (C-6) (Figure S12); DEPT, COSY, COSY expansion, HMQC and HMQC expansion (CDCl_3_) spectra presented in Figures S13–S17.

Synthesis of bis(1-*O*-ethyl-2,3,4,6-tetra-*O*-benzoyl-β-D-galactopyranosyl)amine, **9**.

Prior to reaction, UOP type 3 Å molecular sieves, *N*-Boc-diethanolamine **5** and silver sulfate (protected against light) were dried in a Büchi Glass Oven B-580 under vacuum at 70 °C for at least three hours. Then, *N*-Boc-diethanolamine **5** (0.05 g, 0.25 mmol) solution in DCM (3 mL) and powdered molecular sieves were added while stirring in argon atmosphere. After 10 min, the reaction flask was protected against light and silver sulphate (0.47 g, 1.51 mmol) was added. After 10 min, a DCM (2 mL) solution of the bromide **7** (0.67 g, 1.0 mmol) was added dropwise over 15 min. Upon completion, the reaction mixture was filtered over celite, concentrated and purified by flash column chromatography (2:1 → 1:1 → 1:2 hexane/AcOEt → AcOEt), giving **9** (0.12 g, 38.1%) as a white foam. Koenings-Knorr reaction between 7 and 5, was attempted using different promoters and times. The conditions studied and the products yields obtained are presented in Table S1, at Supplementary Materials.

Rf = 0.5 (AcOEt); FTIR (KBr, cm^−1^), υ: 3422 (N–H st, C-H st), 1729 (C=O), 1636 (Ar), 1315, 1268 (CO–O as st, O–C–C as st), 1109 (C–O–C as st), 1069 (C–O–C sym st) (Figure S18); m.p. 100–104 °C; ${\left[\alpha \right]}_{D}^{27\;{}^{\circ}C}\;=$ +76.06 (c 1, CHCl_3_); ^1^H NMR (400 MHz, CDCl_3_) δ 8.13–7.17 (m, 40H, Ar), 6.00 (s, 2H, 2xH-4), 5.77 (t, *J* = 9.1 Hz, 2H, 2xH-2), 5.61 (d, *J* = 10.6 Hz, 2H, 2xH-3), 4.73 (d, *J* = 7.9 Hz, 2H, 2xH-1), 4.68 (m, 2H, 2xH-6_A_ ), 4.43 (m, 2H, 2xH-6_B_), 4.31 (t, *J* = 6.2 Hz, 2H, 2xH-5), 3.92–3.81 (m, 2H, CH_2_–CH_2_A), 3.49 (d(d, *J* = 4.5 Hz, 2H, CH_2_–CH_2B_), 2.68 (s, 4H, 2xCH_2_–CH_2A+B_) (Figure S19); ^13^C NMR (101 MHz, CDCl_3_) δ 166.1, 165.6, 165, 5, 165.2 (CO), 133.6, 133.4, 133.3, 133.3, 130.0, 129.8, 129.7, 128.6, 128.5, 128.4, 128.3 (C-Ar), 129.4, 129.3, 129.0, 128.8 (Cq-Ar), 101.8 (C-1), 71.6 (C-3), 71.3 (C-5), 69.8 (C-2, CH_2_CH_2_NH), 68.1 (C-4), 62.0 (C-6), 48.6 (CH_2_CH_2_NH) (Figure S20); DEPT, COSY, HMQC, HMBC and NOESY (CDCl_3_) spectra presented in Figures S21–S29; MALDI-TOF MS m/z calcd. for C_72_H_64_NO_20_ (M+) 1262.4016; found: 1262.2275 (Figure S30); ESI-FIA-TOF MS (EM): m/z calcd. for C_72_H_64_NO_20_ (M+) 1262.4016; found: 1262.4017 (Figure S31).

Synthesis of bis(1-*O*-ethyl-β-D-galactopyranosyl)amine, **3a**.

The benzoylated compound 9 (0.160 g, 0.13 mmol), was dissolved in methanol (3 mL) with vigorous stirring in argon atmosphere. Methanolic solution of sodium methoxide was added dropwise (0.5 M, 2 mL). Upon completion (2 h), Dowex 50 W (H+, 180 mg) was added and after 30 min the mixture was filtered and evaporated to dryness. The residue was dissolved in water and washed three times with DCM. The aqueous layer was evaporated to dryness to give **3a** as a white-yellowish solid (0.049, 91% without purification).

FTIR (KBr, cm^−1^), υ: 3384 (O–H and N–H stretch), 2940, 2883 (C–H), 1647 (N–H) (Figure S32); m.p.: 82 °C (decomposition); ${\left[\alpha \right]}_{D}^{18\;{}^{\circ}C}=-8$ (c 0.35, H_2_O); ^1^H NMR (400 MHz, D_2_O) δ: 4.43 (d, *J* = 7.8 Hz, 2H, 2xH-1), 4.13–4.02 (m, 2H, 2xO–CH_2-A_), 3.94 (d, *J* = 3.1 Hz, 2H, 2xH-4), 3.87–3.64 (m, 10H, 2xH-3, 2xO–CH_2-B_, 2xH-_6A_, 2xH-_6B_, 2xH-5), 3.60-3.49 (m, 2H, 2xH-2), 2.95 (t, *J* = 5.0 Hz, 4H, 2xCH_2_) (Figure S33); ^13^C NMR (101 MHz, D_2_O) δ: 102.9 (C-1), 75.2, 72.7 (C-3, C-5), 70.8 (C-2), 68.7 (C-4), 68.1 (O–CH_2_), 61.0 (C-6), 47.6 (HN–CH_2_) (Figure S34); ESI-FIA-TOF MS (EM): m/z calcd. for C_16_H_32_NO_12_ (M+) 430.1919; found: 430.1925 (Figure S35); DEPT, COSY, HMQC and HMBC (CDCl_3_) spectra presented in Figures S36–S39.

Synthesis of bis(1-*O*-ethyl-β-D-galactopyranosyl)amine-PLGA conjugate (PLGA-di-GAL), **10**.

Bis(1-*O*-ethyl-β-D-galactopyranosyl)amine **3a** (0.010 g, 0.02 mmol) was dissolved in dimethylformamide (DMF) (10 mL) in argon atmosphere with magnetic stirring. Both PLGA (200 mg) and the catalyst (20 mg of montmorillonite K10 or 0.02 mL of methanosulfonic acid) were added and the reaction was heated up to 60 °C for 24 h. Then, the reaction mixture was poured into cold water and stored in the fridge for 30 min. The polymer was filtrated, washed with cold water, and dried to afford the polymer conjugate 10 (85% *w*/*w* using montmorillonite K10 clay or 89% using methanosulfonic acid as a catalyst, calculated from the polymer weight).

FTIR (KBr, cm^−1^): 3441 (OH st), 2997 (C–H), 1761 (C=O st) (Figure S40); ^1^H NMR (400 MHz, D_2_O) d: 5.41–5.09 (m, 1H, CH-PLGA), 5.01–4.52 (m, 2H, CH_2_-PLGA), 4.48–4.00 (m, 0.1H, carbohydrate signals), 1.81–1.39 (m, 3H, CH_3_-PLGA) (Figure S41).

### 2.4. Production of PLGA and PLGA-di-GAL Nanoparticles

The synthesized polymer, PLGA-di-GAL, was used to prepare doxorubicin-loaded NP. NP of PLGA were also produced as control. Polymeric NP of each type of polymer (PLGA, PLGA-di-GAL) were prepared using an oil-in-water (*o*/*w*) emulsion method: 80 mg of polymer were dissolved in 4 mL of DCM already containing DOX 1.7 mM. This organic solution was poured into 15 mL of an aqueous solution of PVA 3% *w*/*v* as a stabilizer and sonicated for 20 seconds twice (10 seconds of pausing, 30 W). The o/w emulsion was poured into a solution of PVA 0.25% *w*/*v* and magnetically stirred overnight at room temperature for complete extraction/evaporation of the solvent and NP harden. The produced NP were repeatedly centrifuged at 9000 rpm (Sartorius, Goettingen, Germany) and washed with deionized water and then lyophilized (Telstar Cryodos, Terrassa, Spain) and stored in the freezer until their use. Unloaded NP were prepared likewise with no addition of DOX to the polymer solution. For the synthesis of fluorescence rhodamine loaded nanoparticles (RNP), the same procedure was used where rhodamine (150 μL of rhodamine B 4.2 mM) was added instead of DOX. 

### 2.5. Characterization of Nanoparticles

#### 2.5.1. Surface Morphology

The characterization of NP morphology and estimation of NP size was performed by scanning electron microscopy (SEM). Images were obtained with a J JSM 7001 (JEOL, Freising, Germany) field emission scanning electron microscope with an accelerating voltage of 10 kV after chromium-coating the samples fixed onto metallic studs under argon atmosphere.

#### 2.5.2. Particles Size and Zeta Potential

The size and the zeta potential of the produced NP were measured by dynamic light scattering (DLS) performed using Horiba Scientific Nano Particle Analyzer SZ-100 (Horiba, Kyoto, Japan)with 1 mg of particles in 1 mL of filtered (using a 0.45 μm pore size syringe filter) distilled water at a fixed angle of 90° and at 25 °C. Freeze-dried NP were dispersed in a PVA 0.5% (*w*/*v*) aqueous solution, sonicated, washed three times with deionized water, and subjected to appropriate dilution before each measurement. All measurements were made in triplicate.

#### 2.5.3. Fourier Transformed Infrared Spectroscopy (FTIR)

FTIR spectra were obtained on a Shimadzu FTIR spectrometer (Tokyo, Japan) with a high sensitivity pyroelectric detector (DLATGS). Dried nanoparticles were grinded in a mortar with KBr with a 1:100 ratio to prepare pressed pellets. The recovered tablet was analyzed in the region of 400–4000 cm^−1^.

#### 2.5.4. X-ray Diffraction (XRD)

X-ray powder diffraction of NP were carried in a PANalytical X′Pert PRO diffractometer (Eindhoven, The Netherlands), equipped with an X′Celerator detector, in a BraggBrentano geometry, over the 2θ range from 10° to 90° with a step size of 0.033°, using Cu K-alfa radiation (λ = 1.540598 Å).

#### 2.5.5. Thermal Analysis

Differential scanning calorimetry (DSC) measurements were performed on a scanning calorimeter (Setaram DSC 131, Caluire, France) equipped with a thermal analysis data system connected to a cooling system. Samples of 10 mg of NP were placed in aluminum pans, sealed and heated under nitrogen atmosphere two times at a rate of 10 °C per minute from −20 to 80 °C and from 25 to 250 °C.

#### 2.5.6. Drug Encapsulation Efficiency and Load Content

The amount of doxorubicin in nanoparticles was determined by dissolving 5 mg of particles in 5 mL of DCM followed by vortex agitation. DOX was then extracted using 5 mL of deionized water. The mixture was vortexed and sonicated (20 s, 70%, 30 W), the aqueous phase was removed and the organic phase was washed with water two more times, employing vortex and sonication each time. The aqueous phases were combined and the concentration of DOX was measured by spectrophotometry (Amersham Biosciences, Ultrospec 2100 pro, Buckinghamshire, UK) at the wavelength of 480 nm. The encapsulation efficiency was obtained as the ratio between the mass of DOX loaded in NP and the mass of DOX used in NP production. DOX content was calculated as the ratio between the mass of DOX loaded in the NP and the total NP mass.

#### 2.5.7. In Vitro Doxorubicin Release Studies

In vitro release of DOX from PLGA-di-GAL NP was conducted in phosphate buffer media (PBS) at pH 6 (endosome pH) and in phosphate/citrate buffer medium at pH 5 to simulate lysosomes environments. Ten milligrams of NP were dispersed in 7 mL buffer at pH 5 or 6 in closed centrifuge tubes. The tubes were placed at a temperature of 37 °C and a speed rotation of 120 rpm in a horizontal shaker. At time intervals, the tubes were removed and centrifuged at 9000 rpm for 15 min. The supernatants were collected and the amount of DOX released was quantified as described above. The precipitated NP were re-suspended in 7 mL of fresh buffer and placed back in the incubator. DOX release studies with PLGA NP were also performed for comparison.

#### 2.5.8. In Vitro Interaction of Galactose Conjugated NP with HepG2/C3A and HEK293T

##### Cell Culture

Both HepG2/C3A and HEK293T cells were routinely propagated in static conditions. Briefly, D-MEM culture medium (Dulbecco′s Modified Eagle Medium, Gibco) with 1.1 g/L and 4.5g/L glucose, for HepG2 and HEK293T respectively, and GlutaMAX™ was supplemented with 1% (*v*/*v*) pen/strep and 10% (*v*/*v*) FBS (all from Gibco/Invitrogen, Grand Island, NY, USA). Sub-culture was performed by trypsinization when cellular growth reached approximately 70% confluence. Cultures were grown in t-flasks at 37 °C in a humidified atmosphere with 5% CO_2_. The affinity of different NP to HepG2/C3A asialoglycoprotein receptor was investigated by fluorescence microscopy. HEK293T cells were used as negative control. Cell cultures were sub-cultured onto 24-well plates in 1 mL of culture media. 200 μL (1 mg/mL) of NPs suspension were added to each well and incubated at 37 °C for 24 h. Preliminary studies using different incubation times were performed and the results showed that the profile of NP adhesion to cells was reached at 24 h and that does not vary for more prolonged incubation times. After 24 h, the medium was removed and cells were washed twice with culture medium to remove unbound NP, before observation under an inverted fluorescence microscope (DMRB6000, Leica, Wetzlar, Germany). The experiments were performed in triplicates, at least four times.

##### Cell Viability Assays

To assess the cytotoxicity of the NPs, viability assays were performed. HepG2/C3A cells were seeded in 96-well plates in DMEM low glucose culture medium for 24 h in a CO_2_ incubator (5% CO_2_ at 37 °C). After that, doxorubicin-loaded NP were added in a concentration range of 200 µg/mL to 0.38 ng/mL to generate dose-response curves. An assay with no addition of NP was also performed as a control. After 48 h, the PrestoBlue™ Viability Reagent reduction assay (Thermo Fisher Scientific, Waltham, MA, USA) was performed. Media was removed and 100 μL of a 1:9 ratio of PrestoBlue™ reagent in culture medium were added to each well. Cells were incubated with the reagent at 37 °C for one hour. Fluorescent intensity was measured with an excitation wavelength of 560 nm and an emission wavelength of 590 nm in the micro-plate reader Infinite®200 PRO (NanoQuant, Tecan Trading AG, Männedorf, Switzerland). Fluorescent intensity for each concentration of NP was normalized as a percentage of the fluorescent intensity of the control cells. The experiments were performed in triplicate.

## 3. Results and Discussion

### 3.1. Molecular Modelling

**Figure 2:**
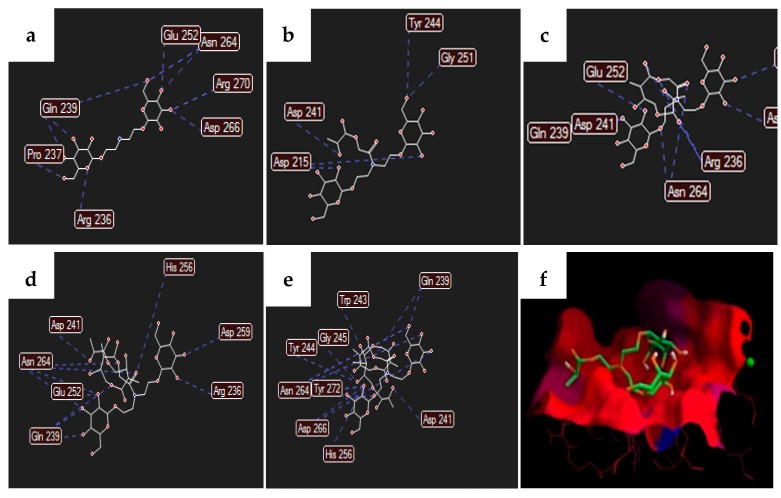
Hydrogen bonds between ASGP-R and ligands (**a**) **3a**, (**b**) **3b**, (**c**) **3c**, (**d**) **3d**, and (**e**) **3e**; (**f**) binding site electrostatic distribution surface with the carbohydrate recognition domain of the H1 subunit of ASGP-R including ligand **3b** in the pocket.

Docking studies of the ligands on the carbohydrate recognition domain of the H1 subunit of the asialoglycoprotein receptor (1DV8 from Protein Data Bank) were performed, using AutoDock Vina, to predict the recognition by the ASGP-R. In order to determine the effect of having a polymer chain attached to the ligand on ASGP-R recognition, since the target compound, PLGA-di-GAL has a long polymer chain, and as it is impossible to apply the full length of the chain in the docking study, the presence of a monomer, a dimer, a trimer and a tetramer as well as the ligands alone (**1a**, **2a**, and **3a**) were analyzed. The ligands studied were the target synthetized compound (**3b**–**e**, Figure 1), with galactose (**1b**–**e**, Figure 1) and *N*-acetylgalactosamine (**2b**–**e**, Figure 1) as controls. The obtained binding energies are presented in Table 1. The main residues of ASGP-R that have shown a greater interaction, either by hydrogen bonding or electrostatic interactions, with the ligand **3a**–**e** are Arg236, Asp241, Gln239, Glu252, and Asn264 (Figure 2 and Figure S1), which is in accordance with the literature [7]. Knowing that galactose (**1a**) and *N*-acetylgalactosamine (**2a**) residues are recognized by ASGP-R, their structures were used as a control. From the binding energies obtained (Table 1), **2a** showed better recognition than galactose (with and without the polymer chain), which is in accordance with the literature [21], with **3a**, the purposed ligand, being the one with the best recognition from ASGP-R.

The addition a monomer and dimer chains (entries 2 and 3) seemed to have a beneficial effect on the binding energies which is due to hydrogen bond interactions between the oxygen of the carbonyl (glycolic region of the polymer) with glutamine 239 residue, and the oxygen of the ester (lactic part) with arginine 236 residue. Figure 2 also shows ligand **3b** interacting with the carbohydrate recognition domain of the H1 subunit of ASGP-R. However, increasing the PLGA chain length to the trimer and tetramer translated in a slight loss of affinity towards the receptor. In fact, the tetramer structures (entry 5) had no significant effect on the recognition compared to the structures without the polymer chain (entry 1). It is clear that, as the polymer chain length increases, the atoms of the polymer involved in hydrogen bond interactions spread apart from the recognition domain, thus not contributing to the binding to the ASGP-R and the binding energies tend to the achieve the same values as the ligands themselves. Regardless the PLGA chain length, the target compound **3a** shows better affinity towards ASGP-R and, therefore, we proceeded with the synthesis of the purposed ligand **3a**.

### 3.2. Synthesis and Characterization of Ligand **3a** and Ligand **3a**-Conjugated PLGA (**10**)

**Figure 3:**
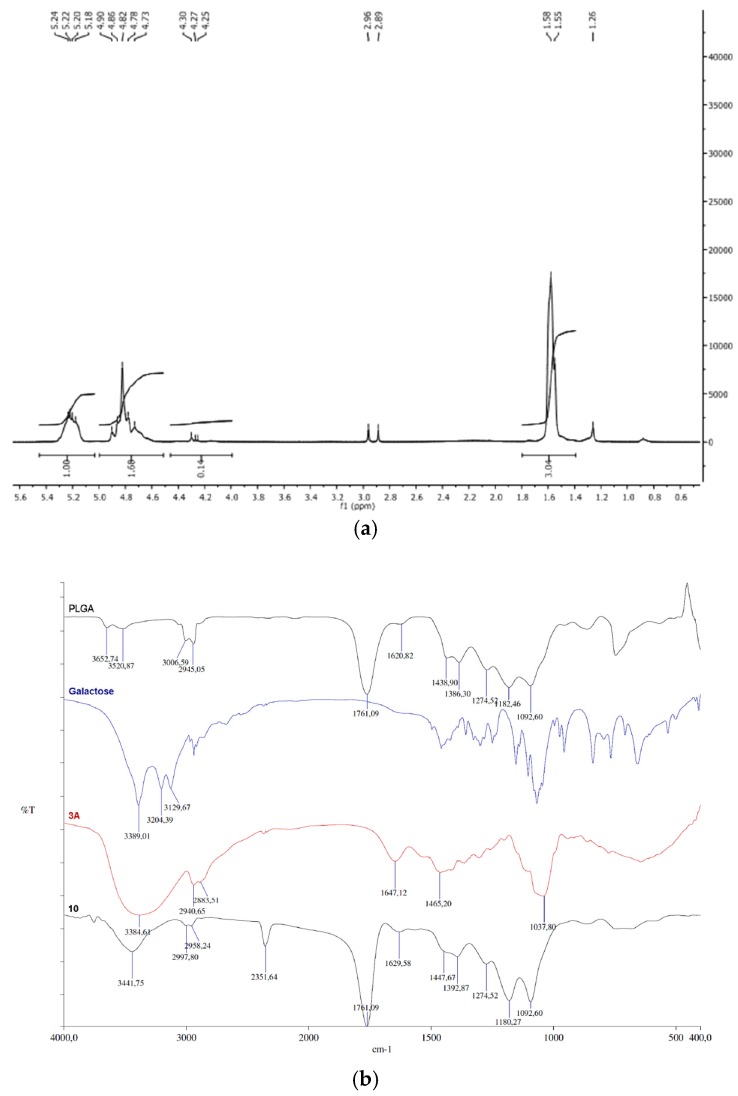
(**a**) ^1^H NMR spectrum (D_2_O) of bis(1-*O*-ethyl-β-D-galactopyranosyl)amine-PLGA conjugate, PLGA-di-GAL, **10**. (**b**) FTIR spectra of PLGA, galactose, bis(1-*O*-ethyl-β-D-galactopyranosyl)amine (**3a**) and PLGA-di-GAL (**10**).

Ligand **3a** and ligand **3a**-conjugated PLGA (PLGA-di-GAL) were synthetized according to Scheme 1. To obtain the desired ligand **3a**, diethanolamine **4** and galactose **1a** were protected with tert-butyloxycarbonyl group as described in the literature [20,22], affording **5** and **7** (Scheme 1), respectively, as proved by NMR spectra (Figures S4–S19 at Supplementary Materials). Koenigs–Knorr conditions between **7** and **5**, using silver sulphate as a promoter, afforded **9** (S20–S33) [20,23,24,25]. More information about other promoters is presented at Supplementary Materials. Proton NMR spectrum of **9** shows that anomeric proton, H-1, has a coupling constant of JH1-H2 = 7.9 Hz, meaning that both H-1 and H-2 are at axial positions, and that the introduced diethanolamine part is at an equatorial position (S19). Further de-*O*-benzoylation of **9**, gave the desired ligand **3a**. Proton and carbon NMR spectra prove that no aromatic protons are present and exact mass analysis confirms the success of the hydroxyls deprotection (S33 and S34). The successfully synthesis of ligand **3a** was also confirmed by FTIR (S32) with peaks at (cm^−1^): 3384 (O–H and N–H stretch), 2940, 2883 cm^−1^ (C–H), 1647 (N–H). Amidation of PLGA with **3a** was attempted using either methanosulfonic acid or montmorillonite K10, following the procedure described in literature [25]. It was found later on that K10 clay is difficult to separate from the final product, thus this method using a zeolite should not be used. The synthetized ligand, **3a**, was further conjugated to PLGA through its carboxylic acid terminal end to get **10**, PLGA-di-GAL. NMR and FTIR spectra of the synthetized ligand **3a** and PLGA-di-GAL are shown in Figure 3. The spectra of galactose and PLGA are also presented for comparison.

The FT-IR spectrum of PLGA-di-GAL is characterized by the presence of the characteristic peak at 1761 cm^−1^, attributed to the ester group of PLGA polymer. Peaks at 3384 cm^−1^ in spectrum of **3a** and at 3441 cm^−1^ in spectrum of **10**, characteristic of OH groups (peaks at 3129, 3204, and 3389 cm^−1^ in the galactose spectrum) are also observed, confirming the successfully synthesis of **3a** and its presence in the PLGA-di-GAL polymer structure. The successfully conjugation of ligand **3a** to PLGA can also be evidenced in the ^1^H-NMR spectrum of PLGA-di-GAL (Figure 3a) where chemical shifts at 1.81–1.39 ppm (3H, –CH_3_), 5.01–4.52 ppm (2H, –CH_2_), 5.41–5.09 ppm (1H, –CH), typical of PLGA, and an additional shift at 4.48–4.00 ppm due to galactose can be seen. Moreover, the yield obtained varied between 84% and 89% which indicates a small loss of PLGA while processing.

### 3.3. Nanoparticle Preparation and Characterization

**Figure 4:**
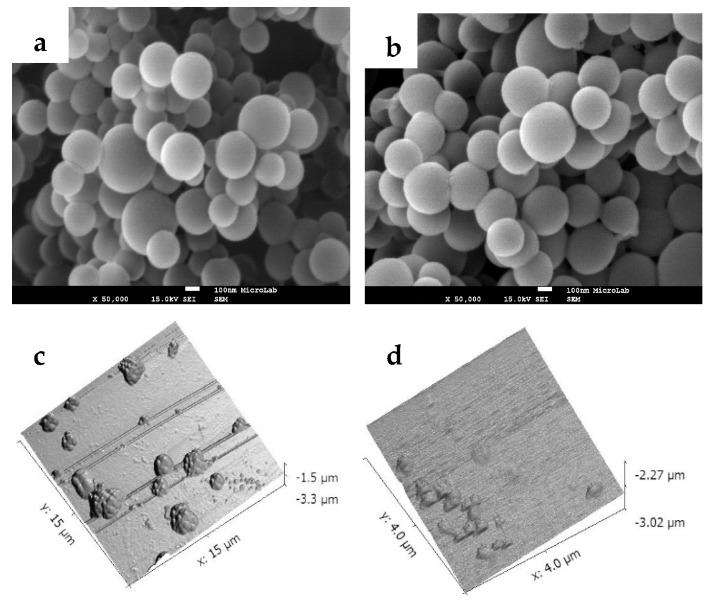
SEM: (**a**) PLGA NP, (**b**) PLGA-di-GAL NP (amplification of 50,000) and AFM: (**c**) clusters of PLGA-di-GAL NP, and (**d**) dispersed PLGA-di-GAL NP.

The produced di-*O*-galactosylated-PLGA polymer, PLGA-di-GAL, was used to prepare unloaded and DOX loaded NP (PLGA-di-GAL NP). Nanoparticles made of commercial PLGA (PLGA NP) were also prepared for comparison. The prepared nanoparticles were characterized in morphology, size, zeta potential, drug load content and crystallinity properties. The surface morphology of PLGA-di-GAL NP was examined by SEM and AFM and representative images are depicted in Figure 4 where a regular spherical shape and a smooth surface (Figure 4b–d) can be observed. SEM of PLGA NP (Figure 4a) is also shown and indicates that the presence of galactose residues on NP surface does not change NP morphology.

SEM image analysis shows PLGA-di-GAL NP with an average diameter of 281 ± 69 nm, in agreement with sizes ranging between 170 and 300 nm obtained by AFM. Some NP clusters with 1–3 µm diameter are visible in AFM images. Zeta potential and size distribution of NP were measured by DLS and the obtained results are presented in Table 2.

Values obtained for PLGA-NP are also presented for comparison. The mean particles diameter obtained, based on intensity (Figure S42) is 311 ± 58 nm with a peak size at 293 nm for PLGA-di-GAL NP and 238 ± 48 nm with a peak size at 211 nm for PLGA NP, respectively. The higher size presented by PLGA-di-GAL NP can be explained by a lower polymer hydrophobicity that can lead to a more unstable o/w interface causing a greater emulsion droplet coalescence during NP preparation. A similar effect was obtained by Peça et al. [26]. A value of −30.3 mV, which shows colloidal stability, was obtained for the zeta potential of PLGA-di-GAL NP at neutral pH indicating the presence of remaining PLGA carboxylic groups. This negative charge may affect the cellular uptake of the prepared nanoparticles due to electrostatic repulsion forces with the negatively charged cell surfaces. However, the introduction of our galactose-based ligand onto the nanoparticles may promote their cellular uptake by receptor-mediated endocytosis in hepatocytes while positively carriers induce a nonspecific interaction with not desirable targets, particularly under in vivo conditions after administration [27]. A DOX encapsulation efficiency of 83 ± 9% was obtained for DOX loaded PLGA-di-GAL NP, equivalent to 3.7 ± 0.4 mg of DOX/100 mg particle while DOX loaded PLGA NP present a value of 65.4 ± 10% equivalent to 2.9 ± 0.4 mg of DOX/100 mg particle. 

**Figure 5:**
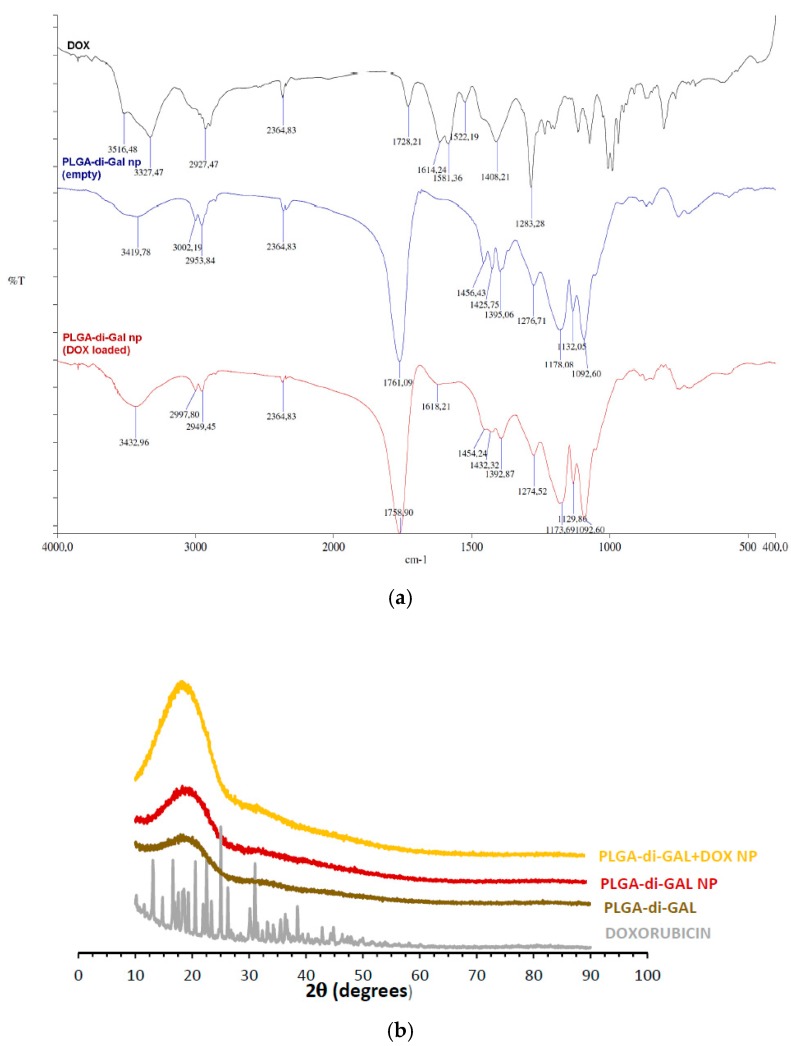
(**a**) FTIR spectra of DOX, empty PLGA-di-GAL NP and DOX loaded PLGA-di-GAL NP; (**b**) X-ray diffraction diagrams of DOX, PLGA-di-GAL polymer, unloaded PLGA-di-GAL NP and DOX loaded PLGA-di-GAL.

Fourier transformed infrared spectroscopy was used to evaluate the chemical structure of DOX loaded PLGA-di-GAL NP. The obtained spectrum is presented in Figure 5a. Spectra of empty PLGA-di-GAL NP and DOX were also added for comparison.

The characteristic peak of PLGA at 1760 cm^−1^ due to the ester group, the bands at 3000–2950 cm^−1^ due to the C–H stretch of CH_3_ and the peaks at 1178–1173 cm^−1^ due to the C–O stretch are present in both loaded and unloaded NP spectra. A band attributed to OH groups at 3419–3433 cm^−1^ can also be seen. The spectrum of DOX loaded PLGA-di-GAL NP present a peak at 1618 cm^−1^, characteristic of the N–H bending vibration of the primary amine present in DOX structure that, as expected, is not present in the unloaded PLGA-di-GAL NP spectrum. The X-ray diffraction spectra of the galactosylated PLGA polymer (PLGA-di-GAL), presented in Figure 5b, demonstrates that the well-characterized amorphous state of PLGA is not affected by the conjugation of a galactose dimer. No peak was also obtained for both empty NP produced using these polymers. This behavior presents an advantage since a more reproducible degradation and kinetic of release are shown by amorphous polymers. Very interesting is the fact that also no peak was obtained for DOX loaded nanoparticles, despite the high crystallinity clearly detected in the spectrum of pure doxorubicin, indicating that doxorubicin is present in the polymer matrix as an amorphous dispersion, which is in accordance with the literature [13]. These results were confirmed by the differential scanning calorimetry thermograms where no melting point was visible therefore indicating an amorphous state of DOX inside PLGA-di-GAL NP. Concerning the polymer glass transition temperature (*T*_g_) (Table 2), results show that is not affected by the presence of two galactose moieties in the PLGA polymer structure.

### 3.4. In Vitro Doxorubicin Release Studies

**Figure 6:**
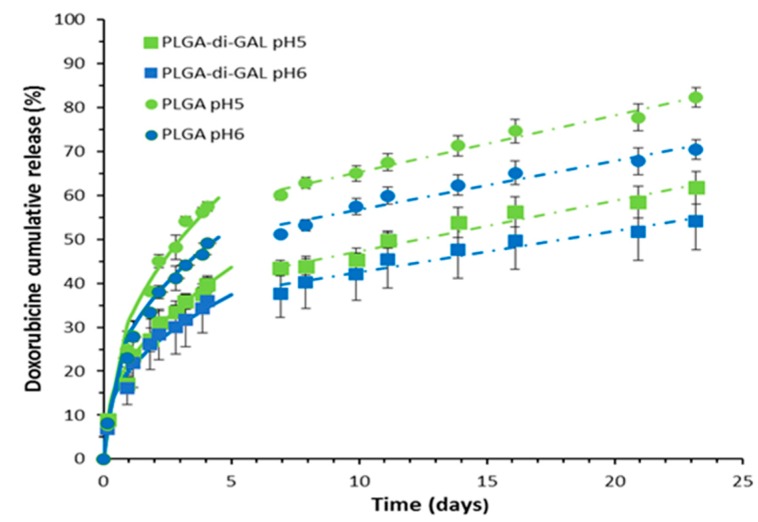
In vitro release of doxorubicin from PLGA–di-GAL NP and PLGA-NP at pH 5 and 6. Data are expressed as the mean ± standard deviation (*n* = 3). Solid lines obtained by the Korsmeyer Peppas model (Equation (1)). Dashed lines obtained by zero order kinetic model.

Figure 6 shows the profile of DOX released from PLGA-di-GAL at pH 5 (close to pH of lysosomes) and pH 6 (close to pH of endosomes) [28,29,30]. The release curve from PLGA NP is also showed for comparison. These pHs were chosen as the recognition of NP by the ASGP-R present in the hepatocyte membrane leads to an interior fold of the membrane, forming a vesicle that evolves into membrane-limited endosome where ligand-receptor dissociation takes place releasing the ASGP-R back to plasma membrane and the ligand into lysosomes, where it is finally degraded and release the drug [8]. Results obtained show that, for both pH under study, PLGA-di-GAL NP present a three-phase release profile: an initial burst release in the first 4 h up to 8.98% for pH 5 and 6.90% for pH 6 presumably due to the drug located on the NP surface that was not removed during NP washing and a sustained release for 25 days.

Such a profile suggests that doxorubicin was primarily encapsulated into the NP matrix rather than adsorbed on NP surface. In order to evaluate the mechanism of drug release and the release rate, the release data obtained were fitted to a semi-empirical equation (Equation (1)) obtained by the introduction of a modification by Kim and Fassihi [31] and Ford et al. [32] that accounts for the burst effect in the Korsmeyer-Peppas model [33]:

(1) $$\frac{M_{t}}{M_{\inf}}-\frac{M_{\mathrm{burst}}}{M_{\inf}}=k\times (t-t_{\mathrm{burst}}{)}^{n}$$

where *M_t_*/*M*_inf_ is the fraction of drug released at time *t*, *M_t_*/*M_burst_* is the fraction of drug released during the burst period (at *t_burst_*), *k* is a kinetic constant characteristic of the network structure, and *n* is a diffusional exponent that indicates the mechanism of transport of a drug through the polymeric matrix. The values of *n* and *k* were obtained fitting the experimental drug release data for *M_t_* < 60% to Equation (1), that is for released fractions obtained until the seventh day, using the simplex algorithms and the least squares minimization of the residuals. The results, within 95% confidence level, are presented in Table 3. The obtained values for the diffusion exponent range from 0.45 to 0.49, showing that the transport of DOX through both nanoparticles matrices until the 7th day, is governed by Fickian diffusion, with the higher values being presented by PLGA-di-GAL NP, for both pH, and by pH 5, for both matrices. The value of *n* found for PLGA-di-GAL NP at pH 5 is slightly higher showing a release mechanism of DOX where Fickian diffusion has a strong contribution but where relaxation polymer chains can occur (0.43 < *n* < 0.85).

From the seventh day onwards, a zero order kinetic release with a constant rate of 1.15% day^−1^ for pH 5 and 0.94% day^−1^ for pH 6 is observed indicating that drug release might be controlled by the polymer dissolution process. The higher rate obtained at pH 5 can be explained by a faster hydrolysis, namely of its ester linkages, at a more acidic environment that increase polymer degradation. PLGA NP also show three drug release phases with higher DOX release rates. Until the seventh day, the release mechanism is also diffusion controlled, after which a zero order kinetic is observed. Results show that a decrease in media pH increase the final amount of DOX released which is a desirable behavior as is in the lysosomes of hepatocytes that the degradation of nanoparticles and the release of DOX will take place. The final amount released results from different physical chemical affinities and interactions between the solute and the polymer matrix or the solvent.

### 3.5. In Vitro Cellular Interactions of Galactose Conjugated NP with HepG2/C3A and HEK293T

**Figure 7:**
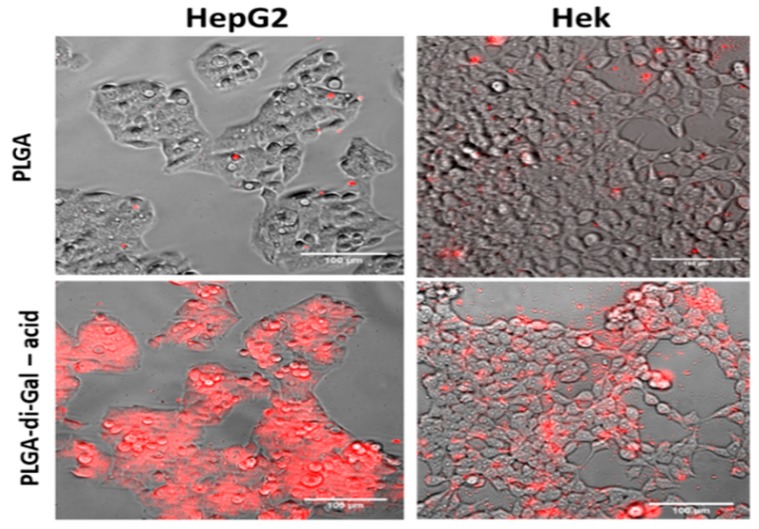
HepG2/C3A and HEK 293T cells after 24 h incubation with different NPs loaded with rhodamine B: Images are composites of fluorescence (rhodamine, red) and phase contrast images. The bars represent 100 µm.

Cell cultures of HepG2/C3A (ASGP-R positive) and Hek293T (ASGP-R negative) were exposed to nanoparticles and assessed by fluorescence microscopy after 24 h, to evaluate the specificity of NP-cell interactions (Figure 7). For a better visualization under fluorescence microscopy, PLGA NP and PLGA-di-GAL NP containing rhodamine B were used in the recognition tests performed.

HepG2 cells incubated with PLGA-di-Gal NP presented detectable red fluorescence in the majority of the cells, indicative of adhesion/internalization of the NP while low adhesion/internalization was observed for HEK 293T suggesting a specific interaction of PLGA-di-Gal particles with hepatic cells. Additionally, it can be observed that non-functionalized NP were not internalized by HepG2/C3A cells leading to the conclusion that the ligand containing two galactose residues on NP surface does promote the recognition of PLGA-di-GAL NP by hepatocyte cells, in a process where ASGPR, expressed in these cells, that has the ability to recognize galactose residues can play a significant role.

### 3.6. In Vitro Cytotoxicity

**Figure 8:**
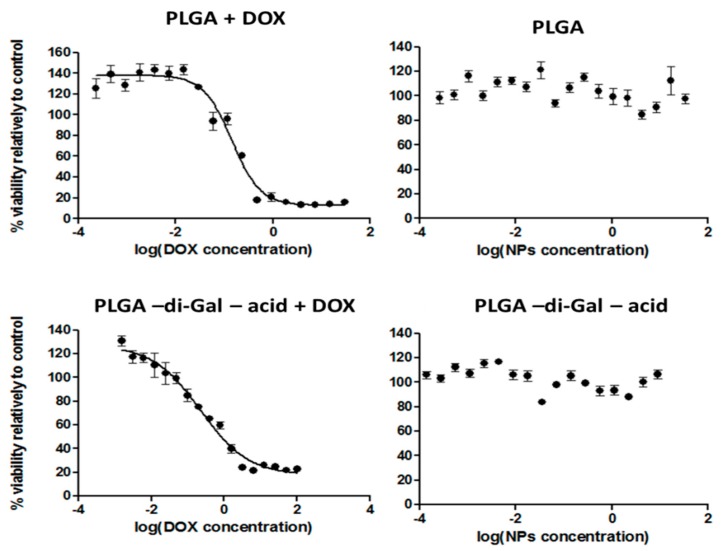
Dose-response curves of doxorubicin-loaded NPs and empty NPs exposed to HepG2 cells for 48h: PLGA NP (30 µg/mL to 0.38 ng/mL); PLGA-di-GAL- acid NP (150 µg/mL to 1.1 ng/mL).

The cytotoxicity of the nanoparticles was determined in vitro in HepG2 cells, measured by the PrestoBlue assay (Figure 8). 

Cell viability after 48 h of incubation with the different unloaded NP formulations was high, around 100% in the range of concentrations tested (Figure 8, right panel), therefore, showing that all produced unloaded NP do not present any toxicity, exhibiting thereof an excellent biocompatibility. In contrast, when HepG2 cells were exposed to NP loaded with DOX, cell viability decreased with increasing concentration of DOX-loaded NP (Figure 8, left panel), in a dose-dependent manner. The half maximal inhibitory concentration (IC50) was determined for each type of DOX loaded NP and value of 0.14 µg/mL and 0.22 µg/mL were obtained for PLGA NP and PLGA-di-GAL NP, respectively. Besides the higher IC50 value presented by PLGA-di-GAL NP, the observed recognition of these NP by Hep G2 cells in relation with cells that do not express the ASGPR offers the ability to greatly reduce the side effects to healthy tissues and may lead to significant improvement in cancer treatment efficacy.

## 4. Conclusions

In this paper, we describe the synthesis and characterization of doxorubicin-loaded PLGA nanoparticles conjugated with a new galactose-based ligand for the specific recognition by human hepatoma cellular carcinoma cells (Hep G2). The new targeting compound, containing two galactose units, was selected using molecular docking combined with quantum chemical calculations for modelling and comparing molecular interactions among the carbohydrate recognition domain of the H1 subunit of the asialoglycoprotein receptor and the ligand. The synthesized polymer was used to effectively prepare spherical doxorubicin-loaded NP with a size of 258 nm, a zeta potential of −62.3 mV and a drug encapsulation efficiency of 83%. The in vitro drug release studies show that PLGA-di-GAL NP could act as a controlled delivery system with a release mechanism being governed by Fickian diffusion until the day 7 and by a zero orders release kinetic onwards. In vitro studies of cell viability demonstrated that these carriers are not toxic for cells and that the presence of galactose confers them the capability to effectively recognize HepG2 cells, probably via ASGP-R, allowing to direct the available particles in suspension to target cells, which offers the ability to reduce side effects caused by unspecific drug uptake into healthy tissues. Therefore, the prepared PLGA-di-GAL NP may be used as a potential drug delivery system for the targeted delivery to liver. The obtained results show that molecular docking combined with quantum chemical calculations can represent a promising tool to develop effective targeting drug delivery systems and can be applied to other tissues/organs. 

## Figures and Tables

**Table 1:** Binding energies obtained from docking studies.

Compound	Binding Energy (kcal/mol)		
	N = 1 (Galactose)	N = 2 (*N*-acetylgalactosamine)	N = 3 (bis(1-*O*-ethyl-β-D-galactopyranosyl)amine)
Na	−5.3	−5.4	−6.0
Nb	−6.0	−6.7	−6.5
Nc	−6.0	−6.8	−6.1
Nd	−5.3	−6.2	−5.9
Ne	−5.5	−5.6	−5.9

**Table 2:** Particle size, zeta potential, polymer glass transition temperature (*T*_g_), encapsulation efficiency (EE), and DOX loading content of DOX-PLGA NP and DOX-PLGA-di-GAL NP.

System	Particle Size (nm) ^a^	Zeta Potential (mV) ^a^	*T*_g_ (°C) ^b^	EE (% *w*/*w*) ^c^	DOX Content (% *w*/*w*) ^d^
PLGA			43.32		
PLGA-di-GAL	--	--	44.47	--	--
PLGA NP	238 ± 48	−18.4	45.55	--	--
PLGA-di-GAL NP	311 ± 58	−30.3	44.14	--	--
DOX-PLGA NP	239 ± 43	−71.7	44.9	65 ± 10	2.9 ± 0.4
DOX-PLGA-di-GAL NP	258 ± 47	−62.3	45.69	83 ± 9	3.7 ± 0.4

^a^ measured by DLS; ^b^ measured by DSC; ^c^ encapsulation efficiency calculated as the ratio between DOX content in the particles and the DOX added at the particles preparation; ^d^ calculated as the ratio between DOX content and particles quantity.

**Table 3:** Diffusion exponent n and constant k, according to Equation (1), for DOX release from PLGA-di-GAL NP and PLGA NP at pH 5 and 6.

Particle	pH	*n*	*n* Error (%)	*k* (day^−1^)	*k* Error (%)	*r* ^2^
PLGA-di-GAL NP	pH5	0.494	5.4	14.6	6.2	0.99
	pH6	0.474	4.4	14.5	6.3	0.99
PLGA NP	pH5	0.450	13.1	25.0	8.3	0.99
	pH6	0.446	3.83	19.2	3.0	0.98

## Supplementary Materials

The following are available online at https://www.mdpi.com/2073-4360/12/1/94/s1.
