# Supplementary material for: Development of Novel Galactosylated PLGA Nanoparticles for Hepatocyte Targeting Using Molecular Modelling
Source: Polymers (Basel). 2020 Jan 4;12(1):94. doi: 10.3390/polym12010094 (PMC7023654; doi:10.3390/polym12010094)
Supplement: Supplementary file 1 [file polymers-12-00094-s001.pdf]

Supplementary Material

Development of novel galactosylated PLGA nanoparticles for hepatocyte targeting using molecular modelling

Cláudia D. Raposo[1], Rita Costa[2,3], Krasimira T. Petrova[1,*], Catarina Brito[2,3], Marcus T. Scotti[4] and M. Margarida Cardoso[1,*]

[1] LAQV-REQUIMTE, Departamento de Química, Faculdade de Ciências e Tecnologia, Universidade NOVA de Lisboa, Quinta da Torre, 2829-516 Caparica, Portugal.
[2] iBET, Instituto de Biologia Experimental e Tecnológica, Apartado 12, 2781-901 Oeiras, Portugal.
[3] Instituto de Tecnologia Química e Biológica António Xavier, Universidade Nova de Lisboa, Av. da República, 2780-157 Oeiras, Portugal.
[4] Departamento de Química, Centro de Ciências Exatas e da Natureza, , Universidade Federal da Paraíba Campus I, 58051-900 João Pessoa-PB, Brasil.
* Correspondence: margarida.cardoso@fct.unl.pt; Tel.: +351 212948385; k.petrova@fct.unl.pt

Contents

1. Molecular modelling

2. Synthesis

3. Characterization of compounds (Scheme1)

4. Characterization of nanoparticles

References

1- Molecular modelling

Ligand's 3D structures were energy-minimized using Gamess 12.0 by employing semi-empirical method AM1. Then, a conformational analysis of each ligand was performed in order to select its global minimum, using MMFF method of Spartan 1.2.0. Docking studies were performed using AutoDock Vina. Auto Dock Tools 4.0 was used to prepare the ligands and the macromolecule. The macromolecule used for the studies was 1DV8 from Protein Data Bank, and all the water molecules were removed. Gasteiger charges and polar hydrogen (noBondOrder method) were added to the ligands and non-polar hydrogens were merged.

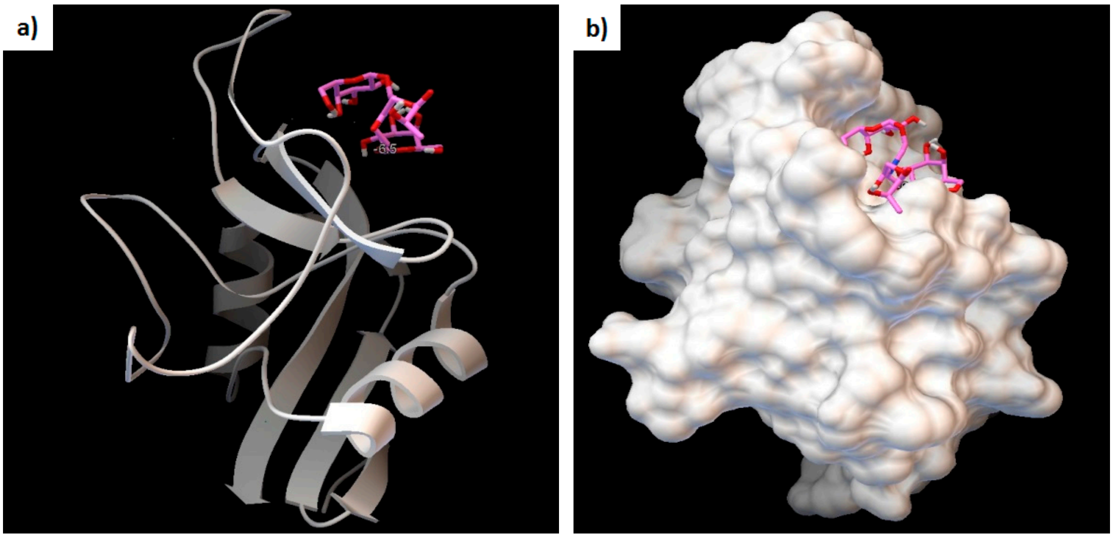

S1. Ligand **3b** interacting with the carbohydrate recognition domain of the H1 subunit of ASGP-R in its a) ribbon diagram and b) surface.

2- Synthesis

Initially, diethanolamine, **4**, is protected with tert-butyloxycarbonyl group (Boc), using the procedure described in the literature [22], affording N-Boc-diethanolamine, **5**, with high yield (89 %, Scheme 1). Galactose, **1**, was also protected using benzyl bromide to afford the stereospecific product, 1,2,3,4,6-penta-O-benzoyl-α-D-galactopyranose, **6**, with 90% yield. Proton Nuclear Magnetic Resonance (1H NMR) spectrum shows the stereospecificity of the reaction, since the anomeric proton at 6.95 ppm is a doublet with J1-2=6.96 Hz, indicating that protons 1 and 2 are both in the axial position, thus benzoyl group being in the equatorial position (β anomer). Meanwhile, Carbon Nuclear Magnetic Resonance (13C NMR) spectrum shows the presence of only one compound because only one signal is present between 80 and 100 ppm, corresponding to one anomeric carbon, meaning that no α anomer is present.

The reaction of **6** with hydrogen bromide leads to the galactosyl bromide **7** very quickly and with high yield (95%), but this product is relatively unstable due to fast bromine hydrolysis, thus not being purified and used right away. Koenigs-Knorr reaction of **7** with previously obtained N-Boc-diethanolamine, **5**, was attempted using different promoters and reaction times, using molecular sieves 3Å as the desiccant agent [20, 23-24]. The conditions used and the products yields obtained are presented in table 1.

Table S1. Promoters used in Koenigs-Knorr condensation.

Entry	Promoter	Number of equivalents	Protected against light	Time (h)	Product	Yield (%)
1	Ag_2CO_3	3	No	17	**8**	73.6
2	Ag_2CO_3 + I_2	3<br>catalytic	Yes	15	**8**	51.0
3	Ag_2CO_3 +<br>I_2	3<br>catalytic	Yes	40	**8**	55.8
4	Ag_2CO_3	6	Yes	17	**8**	98.9
5	Ag_2SO_4	6	Yes	17	**9**	38.1
6	Cs_2CO_3	6	No	16	**none**	N/A
7	$AgCO_2CF_3$	5	No	17	**none**	N/A
8	Bu_4NBr +<br>Na_2CO_3	0.8<br>2	Yes	41	**hydrolysis**	58.6

In the majority of the cases, the reaction mixture was protected from light because of the promoter sensitivity. The desired di-galactopyranosyl product, **9**, was obtained only when silver sulphate was used as the promoter (entry 5). Boc-protecting group of diethanolamine was removed during the reaction, thus its removal using other catalysts was not necessary. According to the literature data, Boc can be removed under basic conditions, which explains this result [34]. Unfortunately, the reaction also leads to the hydrolysis of the galactosyl donor, whereby XX is obtained as a side product. In the cases where silver carbonate was the main promoter (entries 1-4), the mono-galactopyranosyl product, **8**, was obtained. Adding iodine to slow down the hydrolysis reaction did not lead to the desired di-galactopyranosyl product and a reduction of the yield was observed comparing to entry 1 [35]. Cesium carbonate (entry 6) and silver trifluoracetate (entry 7) didn't lead to any product, probably because of low solubility of reactants in the chosen reaction media [35,36]. The reaction with tetrabutylammonium bromide and sodium carbonate (entry 8) [23] lead to hydrolysis of the galactosyl donor, **7**, and only XX was obtained.

3- Characterization of compounds structure (Scheme 1)

N-Boc-diethanolamine, (tert-butyl bis(2-hydroxyethyl)carbamate), 5

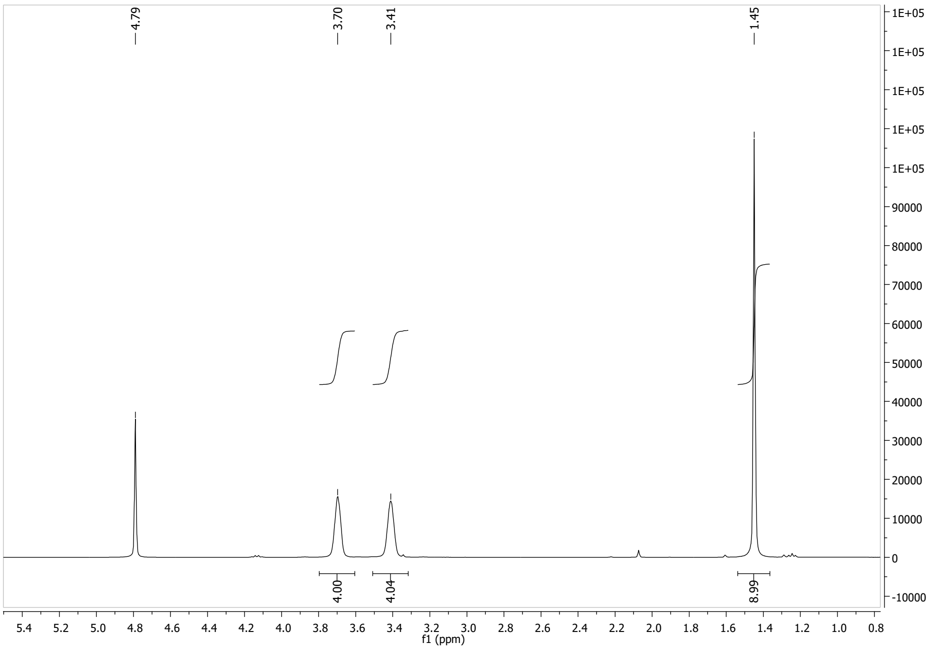

S2. ^{1}H NMR spectrum (D_2O) of *N*-Boc-diethanolamine, (*tert*-butyl bis(2-hydroxyethyl)carbamate), **5**

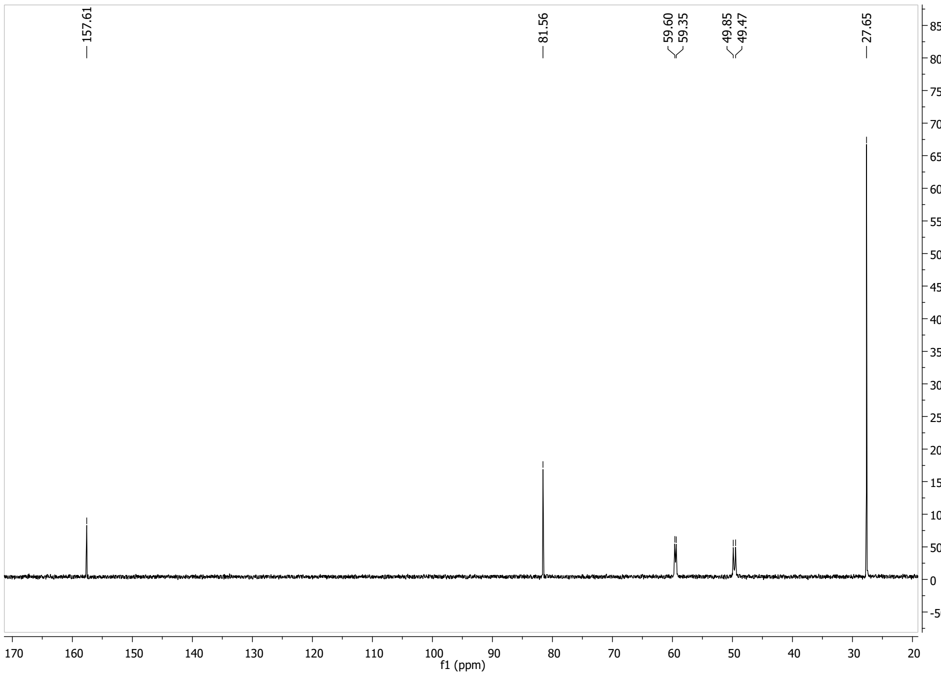

S3. ^{13}C NMR spectrum (D_2O) of *N*-Boc-diethanolamine, (*tert*-butyl bis(2-hydroxyethyl)carbamate), **5**

1,2,3,4,6-penta-O-benzoyl-a-D-galactopyranose, 6

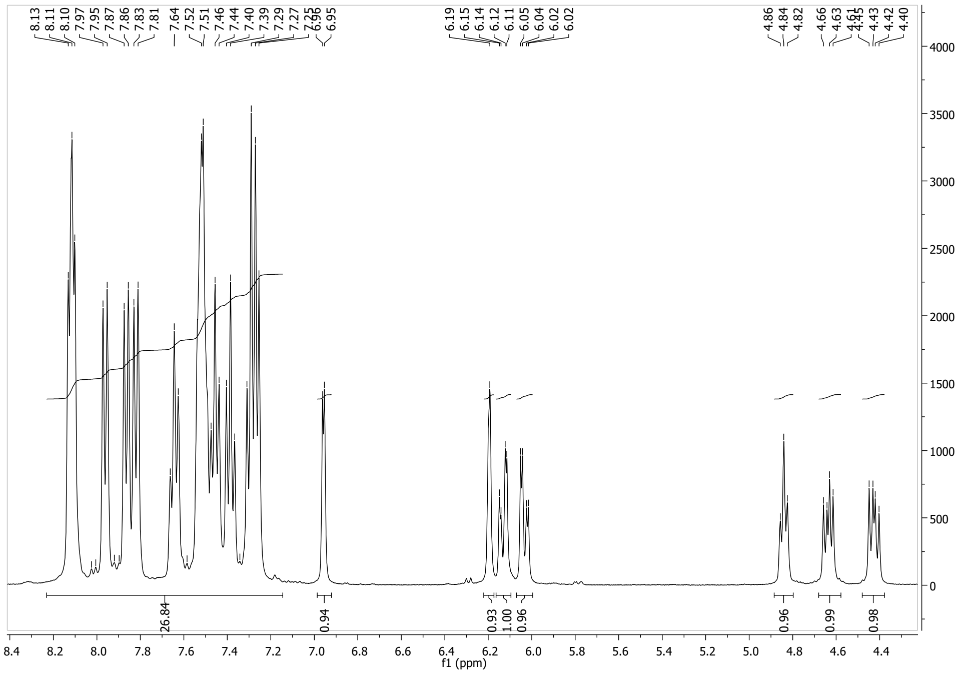

S4. ^{1}H NMR spectrum in ($CDCl_3$) of 1,2,3,4,6-penta-*O*-benzoyl-α-D-galactopyranose, **6**.

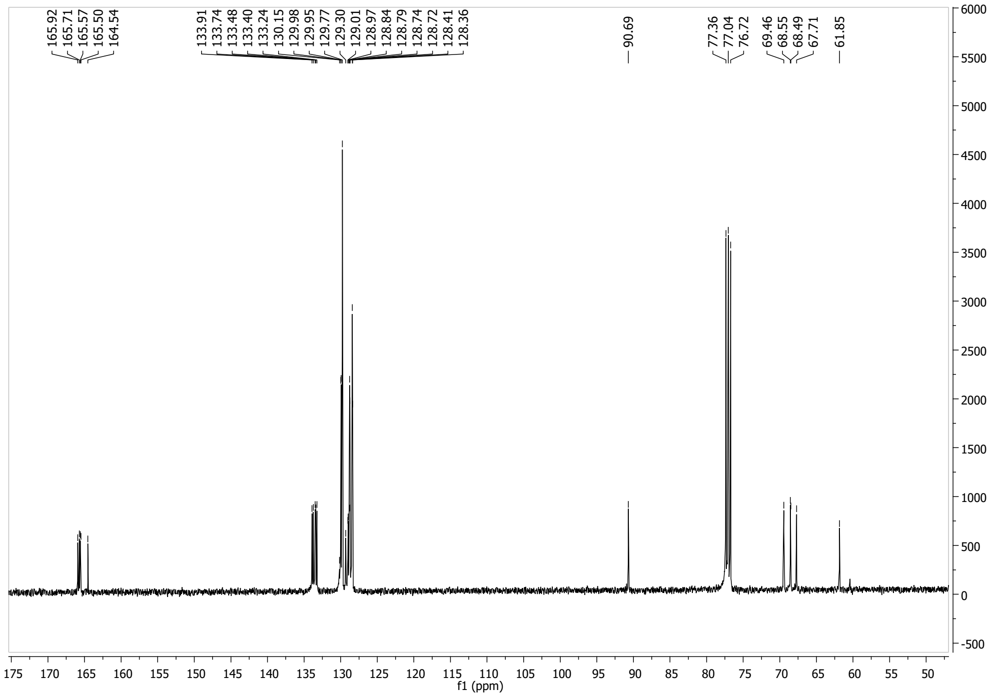

S5. ^{13}C NMR spectrum ($CDCl_3$) of 1,2,3,4,6-penta-*O*-benzoyl-α-D-galactopyranose, **6**.

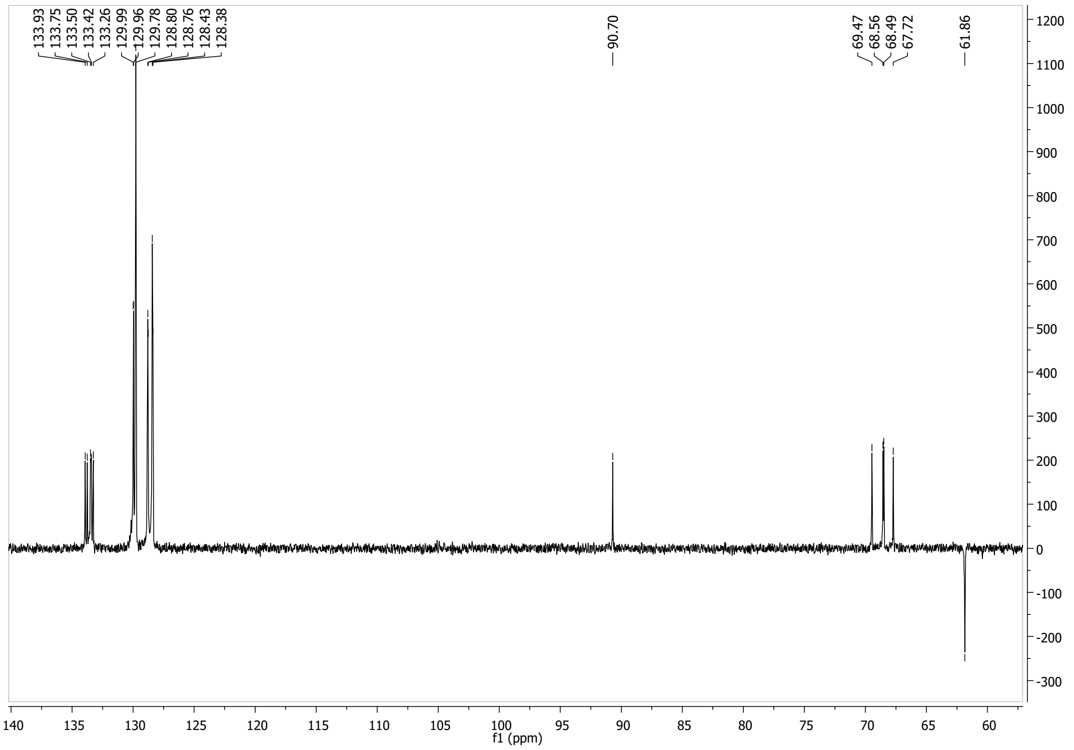

S6. DEPT spectrum ($CDCl_3$) of 1,2,3,4,6-penta-*O*-benzoyl-α-D-galactopyranose, **6**.

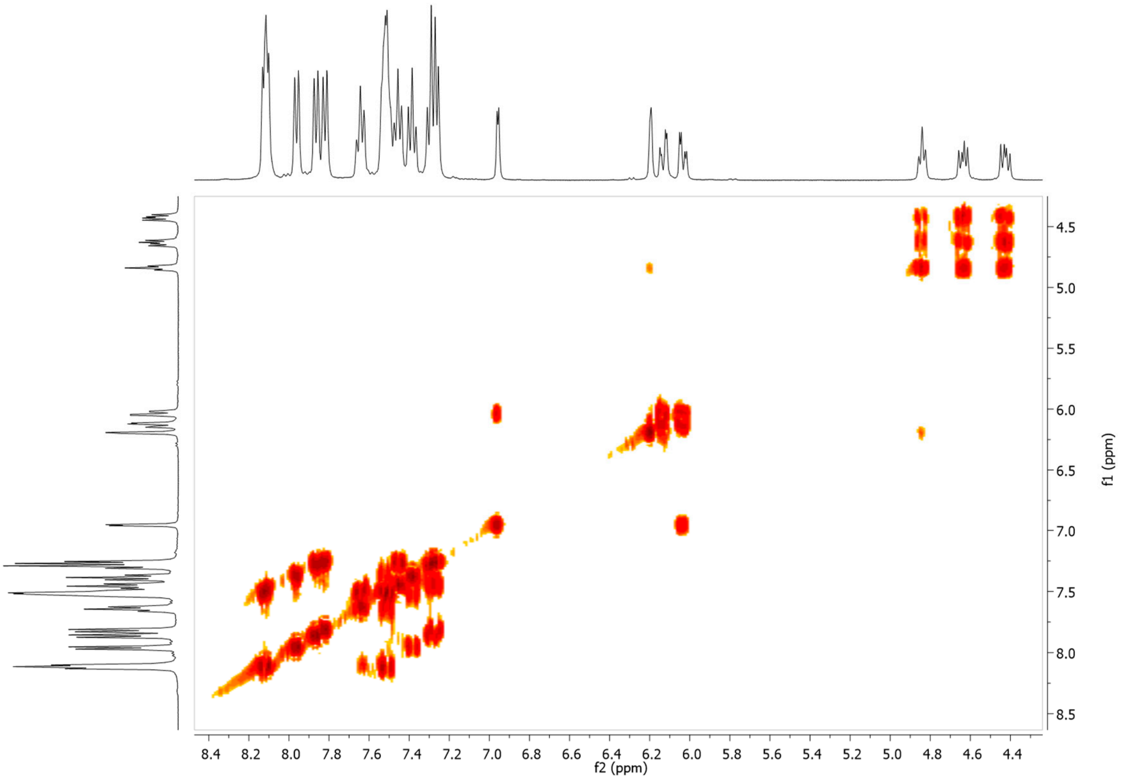

S7. COSY spectrum ($CDCl_3$) of 1,2,3,4,6-penta-*O*-benzoyl-α-D-galactopyranose, **6**.

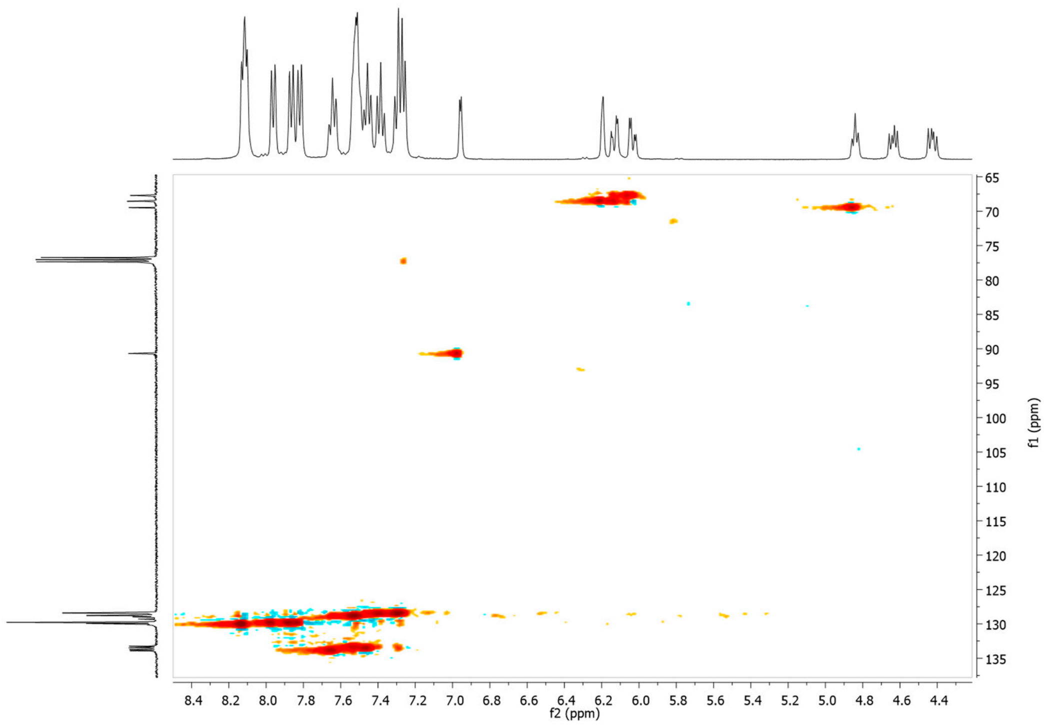

S8. HMQC spectrum ($CDCl_3$) of 1,2,3,4,6-penta-*O*-benzoyl-α-D-galactopyranose, **6**.

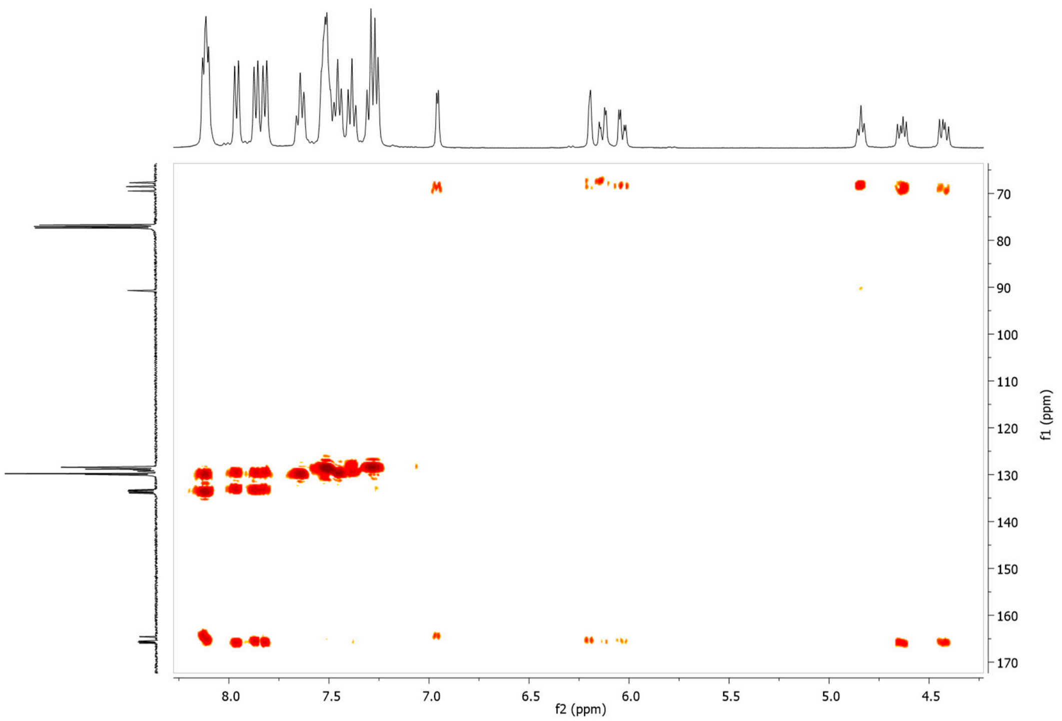

S9. HMBC spectrum ($CDCl_3$) of 1,2,3,4,6-penta-*O*-benzoyl-α-D-galactopyranose, **6**.

2,3,4,6-tetra-O-benzoyl-α-D-galactopyranosyl bromide, 7

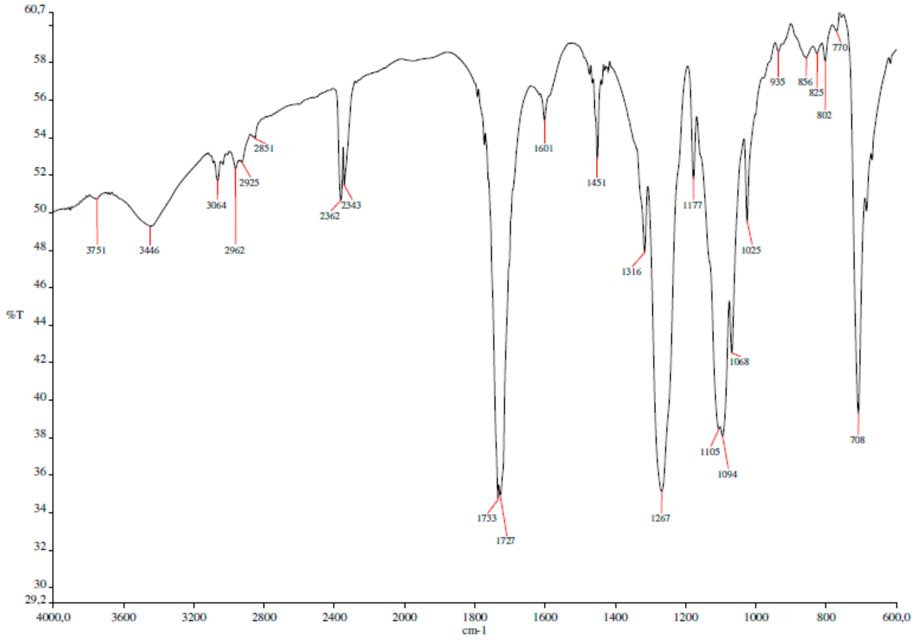

S10. FTIR spectrum (KBr) of 2,3,4,6-tetra-*O*-benzoyl-α-D-galactopyranosyl bromide, **7**.

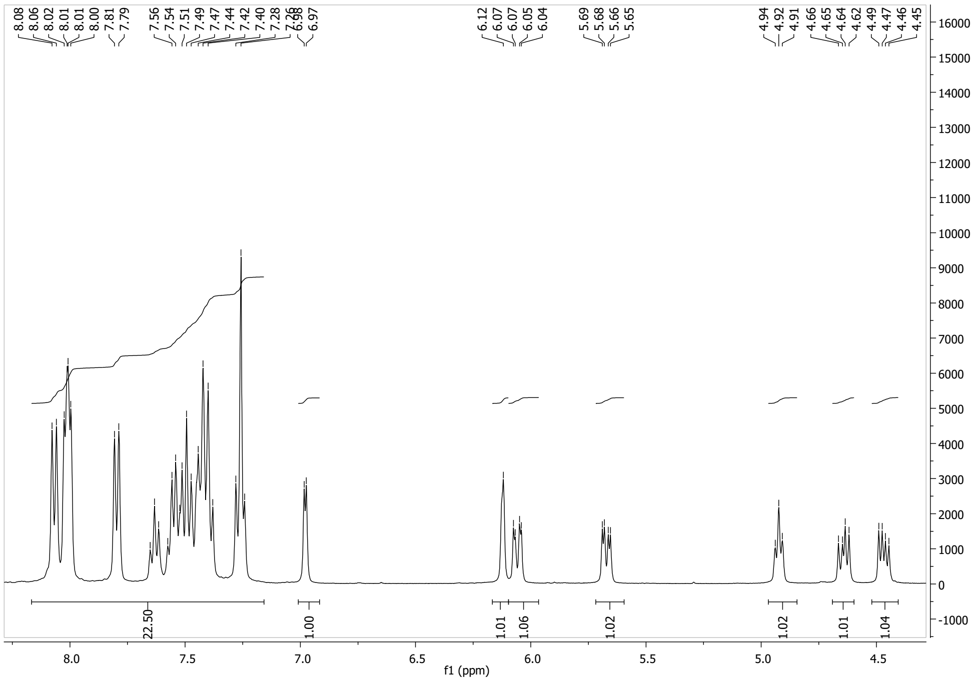

S11. ^{1}H NMR spectrum ($CDCl_3$) of 2,3,4,6-tetra-*O*-benzoyl-α-D-galactopyranosyl bromide, **7**.

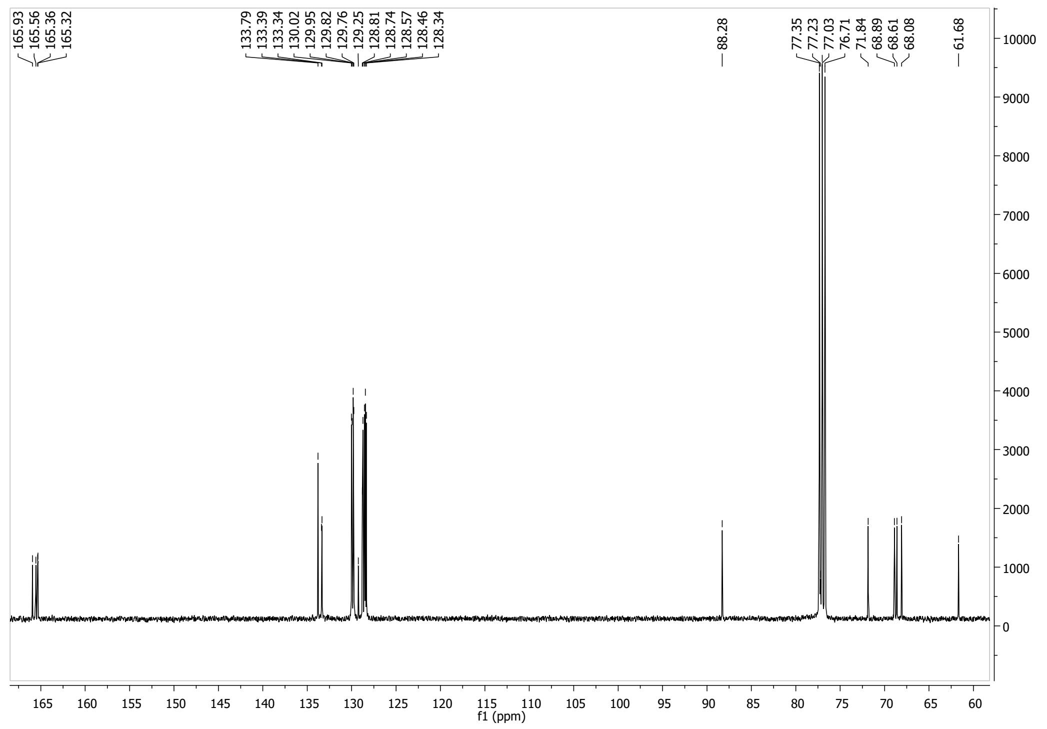

S12. ^{13}C NMR spectrum ($CDCl_3$) of 2,3,4,6-tetra-*O*-benzoyl-α-D- galactopyranosyl bromide, **7**.

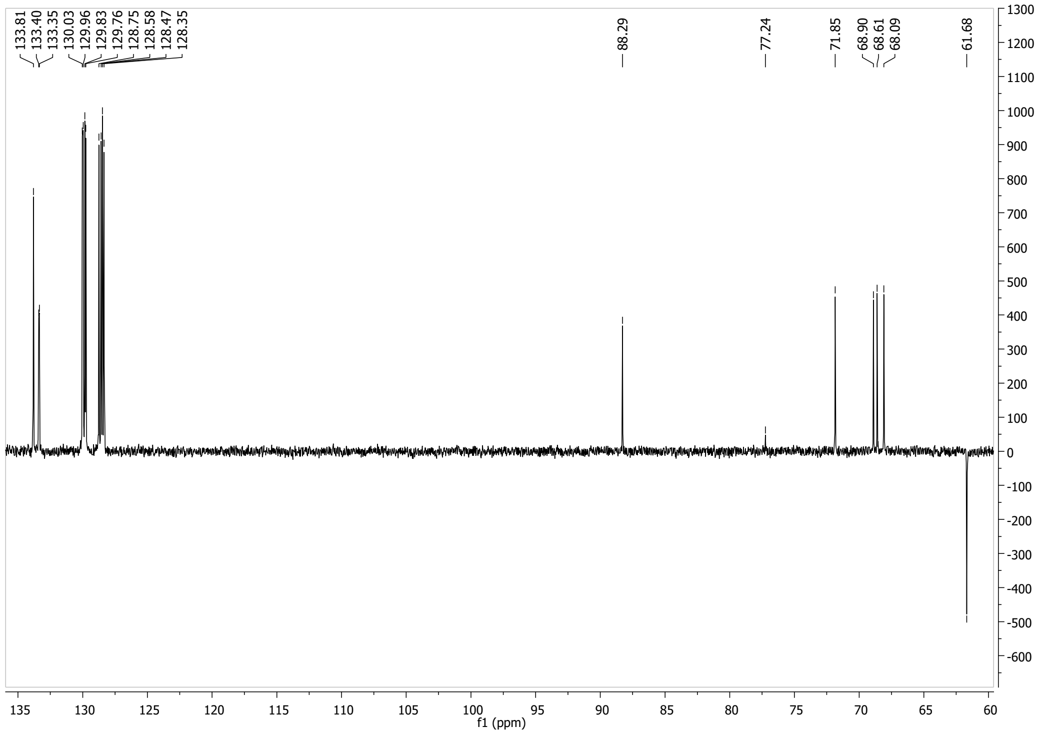

S13. DEPT spectrum ($CDCl_3$) of 2,3,4,6-tetra-*O*-benzoyl-α-D-galactopyranosyl bromide, **7**.

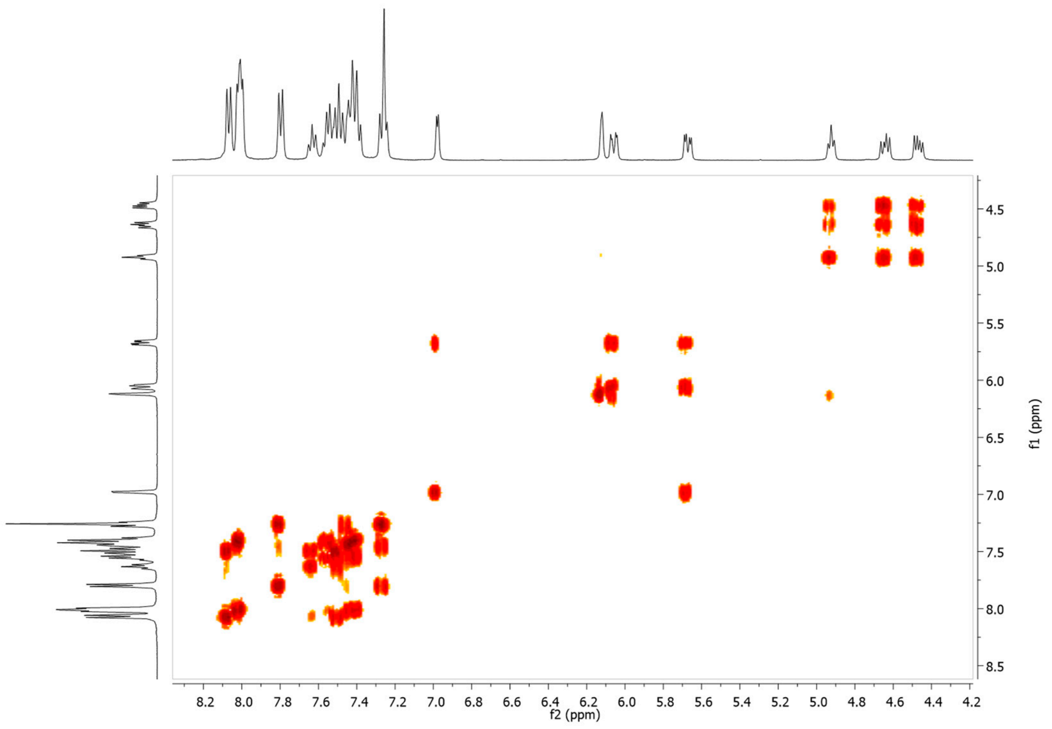

S14. COSY spectrum ($CDCl_3$) of 2,3,4,6-tetra-*O*-benzoyl-α-D-galactopyranosyl bromide, **7**.

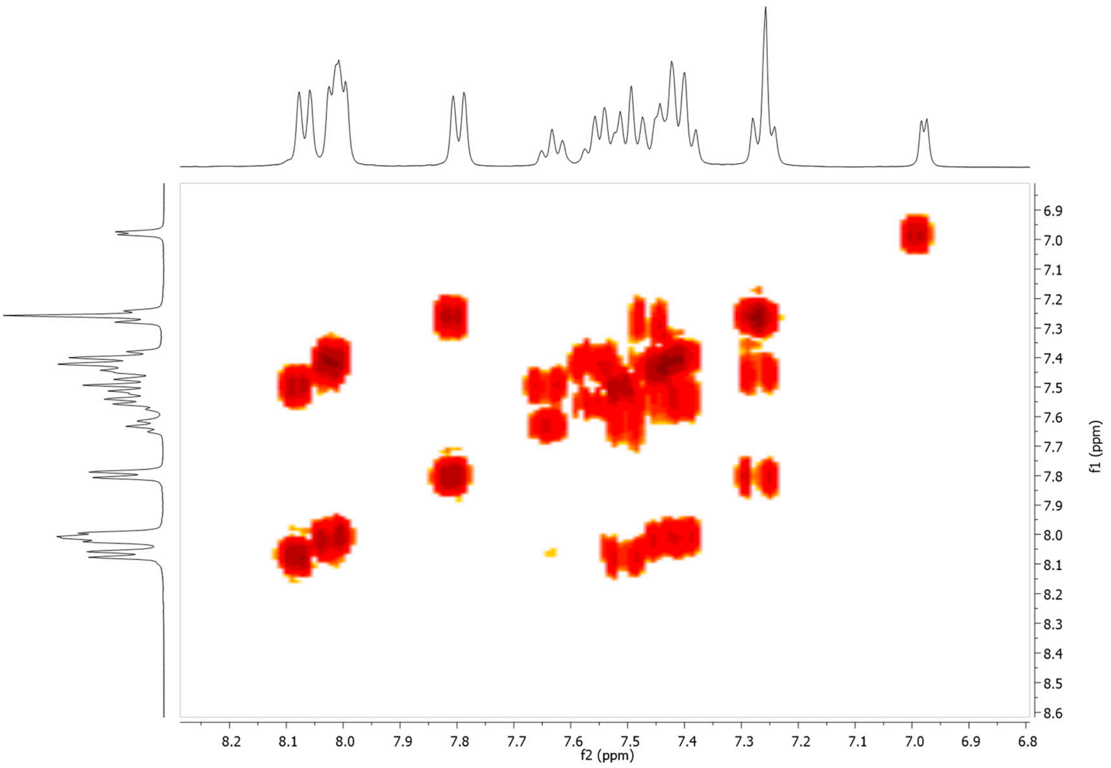

S15. COSY expansion of 2,3,4,6-tetra-*O*-benzoyl-α-D-galactopyranosyl bromide, **7**.

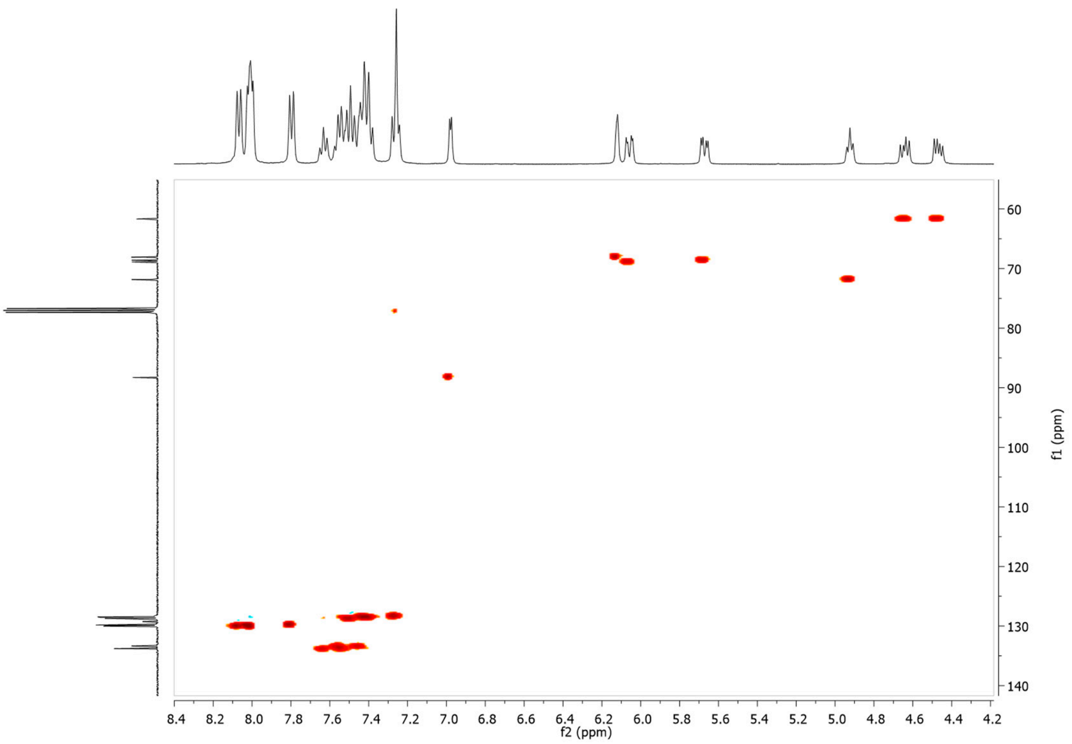

S16. HMQC spectrum ($CDCl_3$) of 2,3,4,6-tetra-*O*-benzoyl-α-D-galactopyranosyl bromide, **7**.

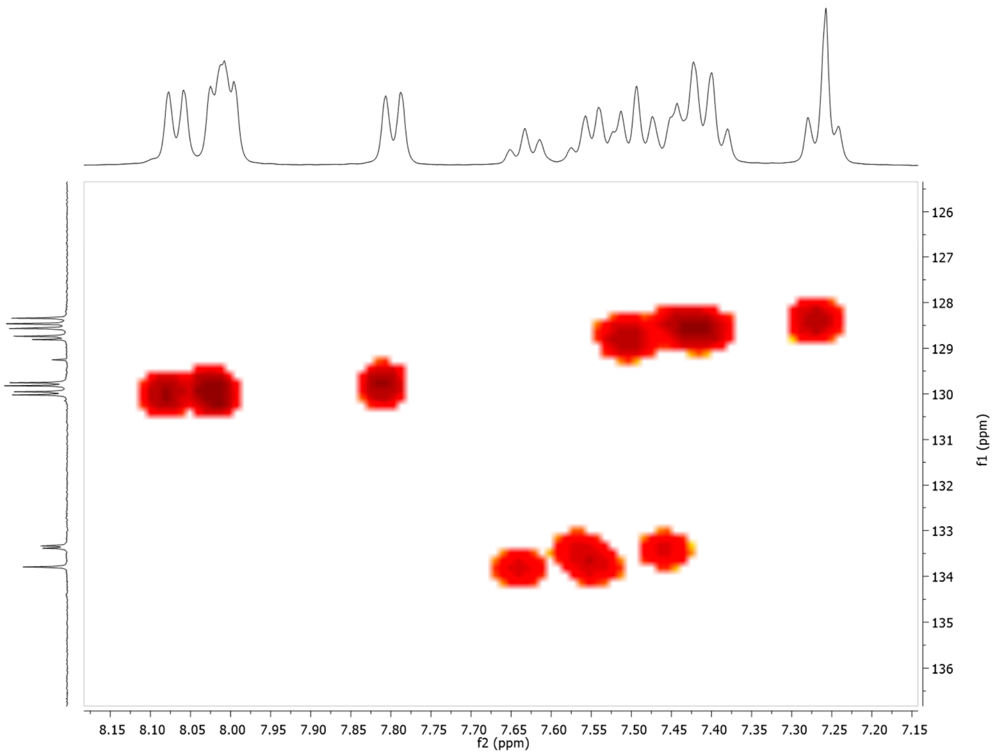

S17. HMQC expansion of 2,3,4,6-tetra-*O*-benzoyl-α-D-galactopyranosyl bromide, **7**.

Bis(1-O-ethyl-2,3,4,6-tetra-O-benzoyl-β-D-galactopyranosyl)amine, 9

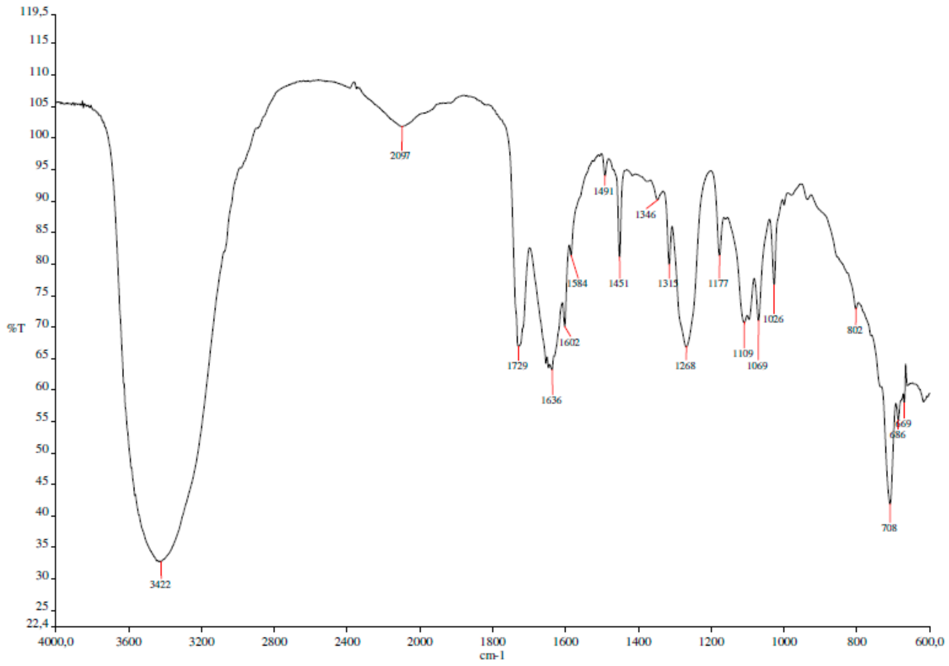

S18. FTIR spectrum (KBr) of bis(1-*O*-ethyl-2,3,4,6-tetra-*O*-benzoyl-β-D-galactopyranosyl)amine, **9**.

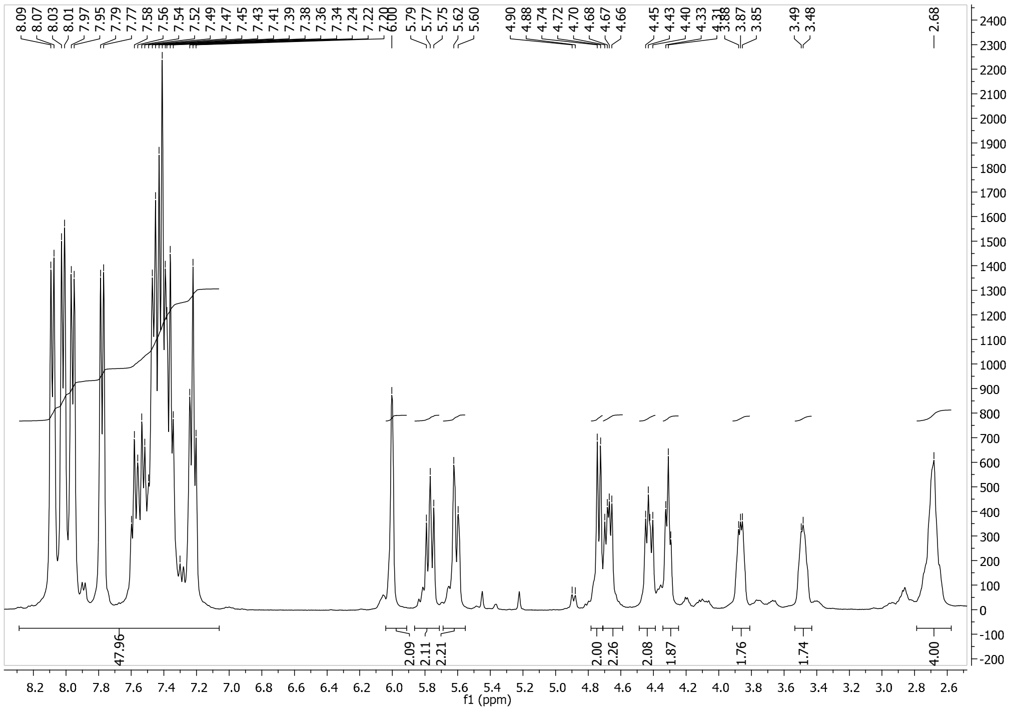

S19. ^{1}H NMR spectrum ($CDCl_3$) of bis(1-*O*-ethyl-2,3,4,6-tetra-*O*-benzoyl-β-D-galactopyranosyl)amine, **9**.

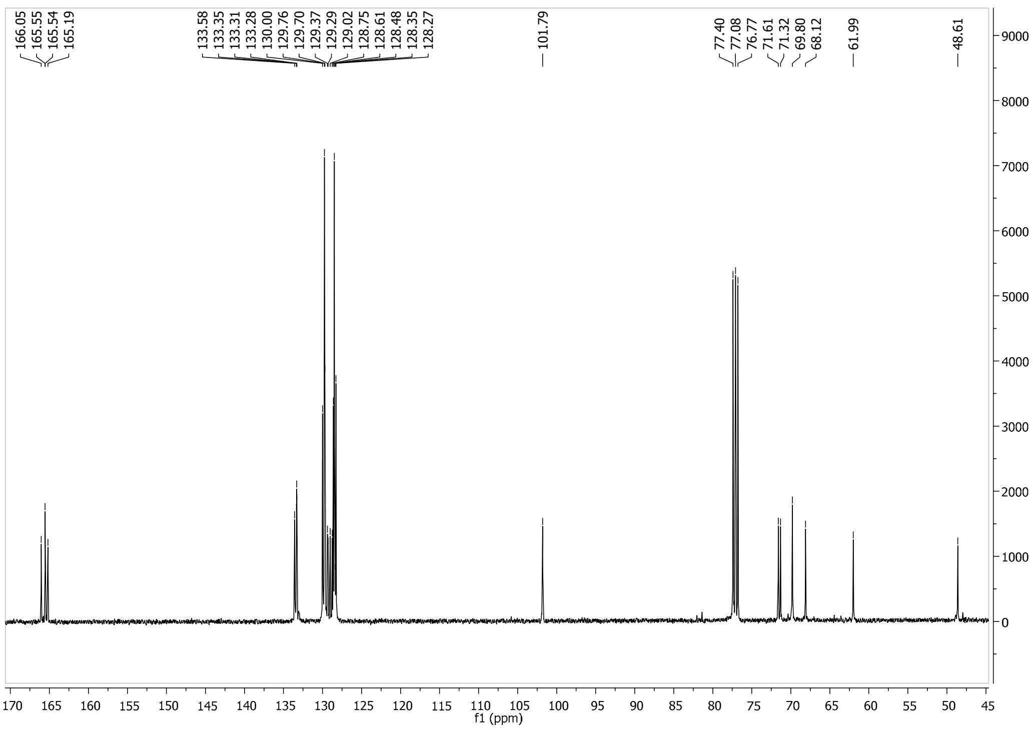

S20. ^{13}C NMR spectrum ($CDCl_3$) of bis(1-*O*-ethyl-2,3,4,6-tetra-*O*-benzoyl-β-D-galactopyranosyl)amine, **9**.

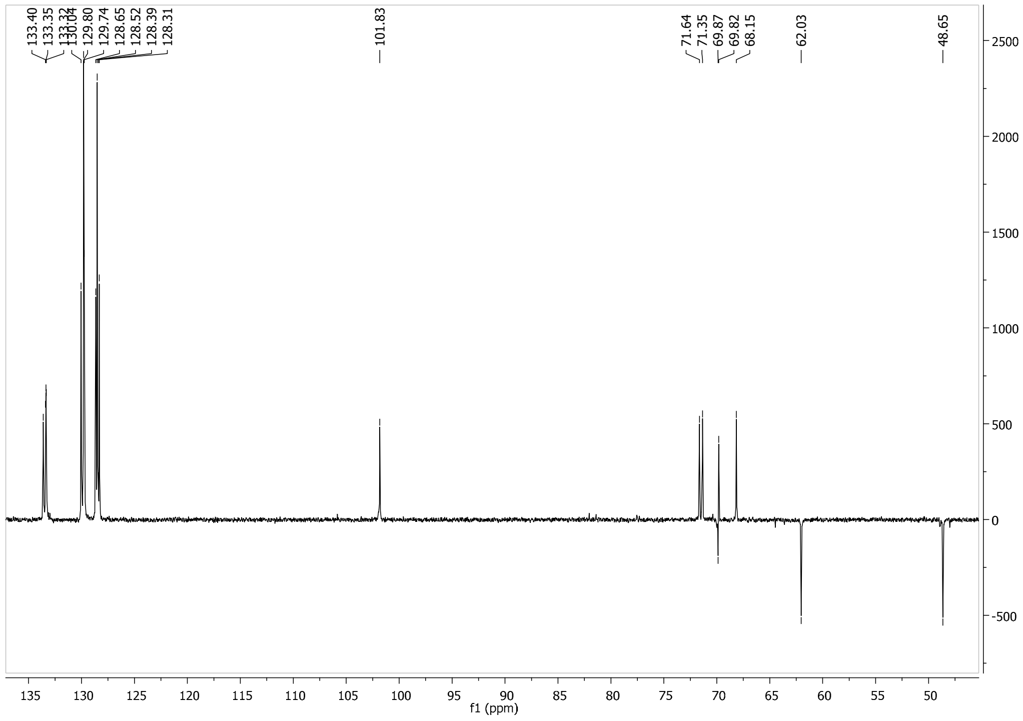

S21. DEPT spectrum ($CDCl_3$) of bis(1-*O*-ethyl-2,3,4,6-tetra-*O*-benzoyl-β-D-galactopyranosyl)amine, **9**.

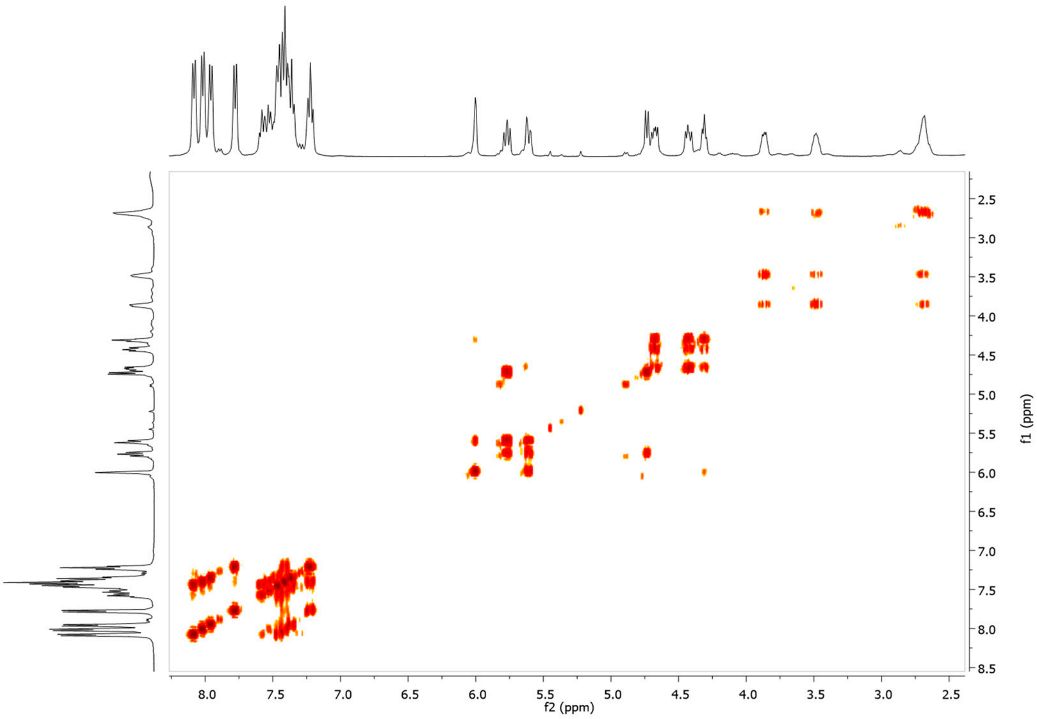

S22. COSY spectrum ($CDCl_3$) of bis(1-*O*-ethyl-2,3,4,6-tetra-*O*-benzoyl-β-D-galactopyranosyl)amine, **9**.

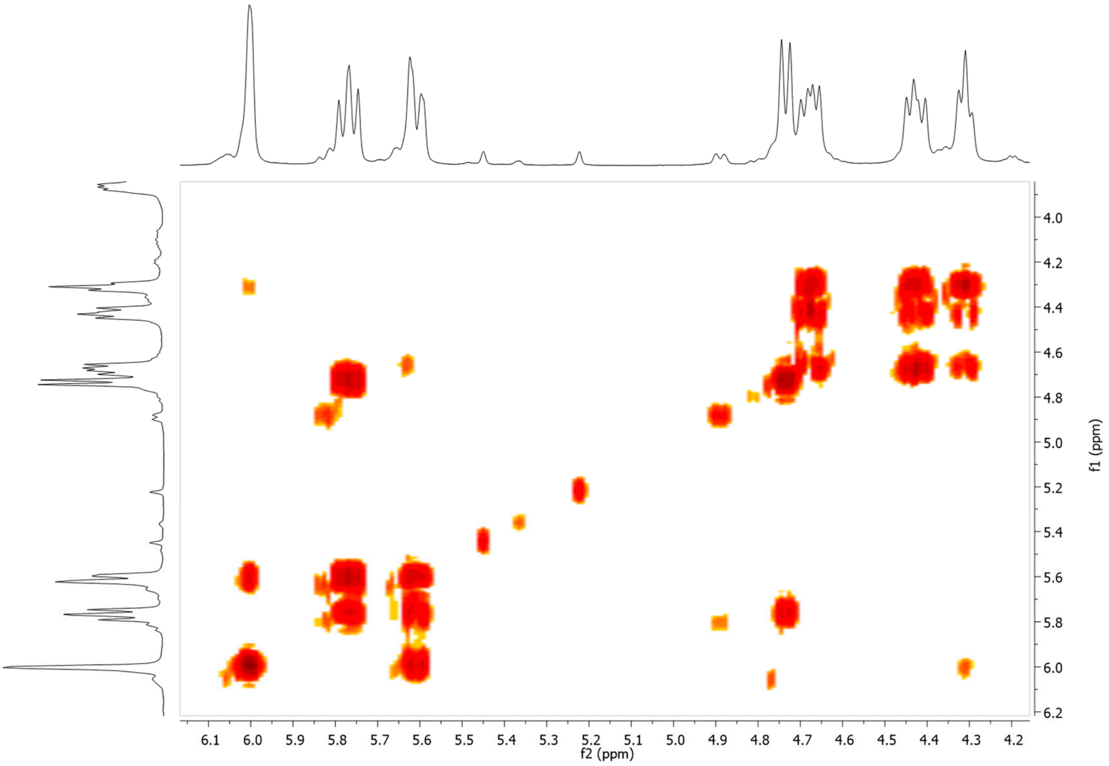

S23. COSY expansion of bis(1-*O*-ethyl-2,3,4,6-tetra-*O*-benzoyl-β-D-galactopyranosyl)amine, **9**.

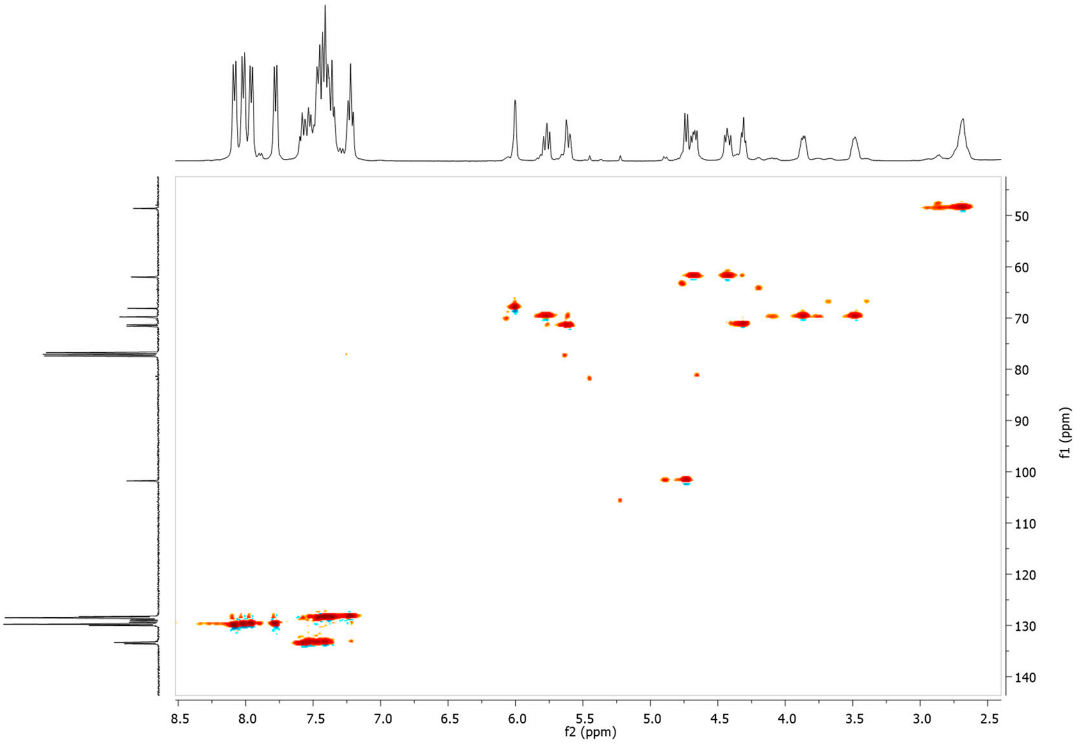

S24. HMQC spectrum ($CDCl_3$) of bis(1-*O*-ethyl-2,3,4,6-tetra-*O*-benzoyl-β-D-galactopyranosyl)amine, **9**.

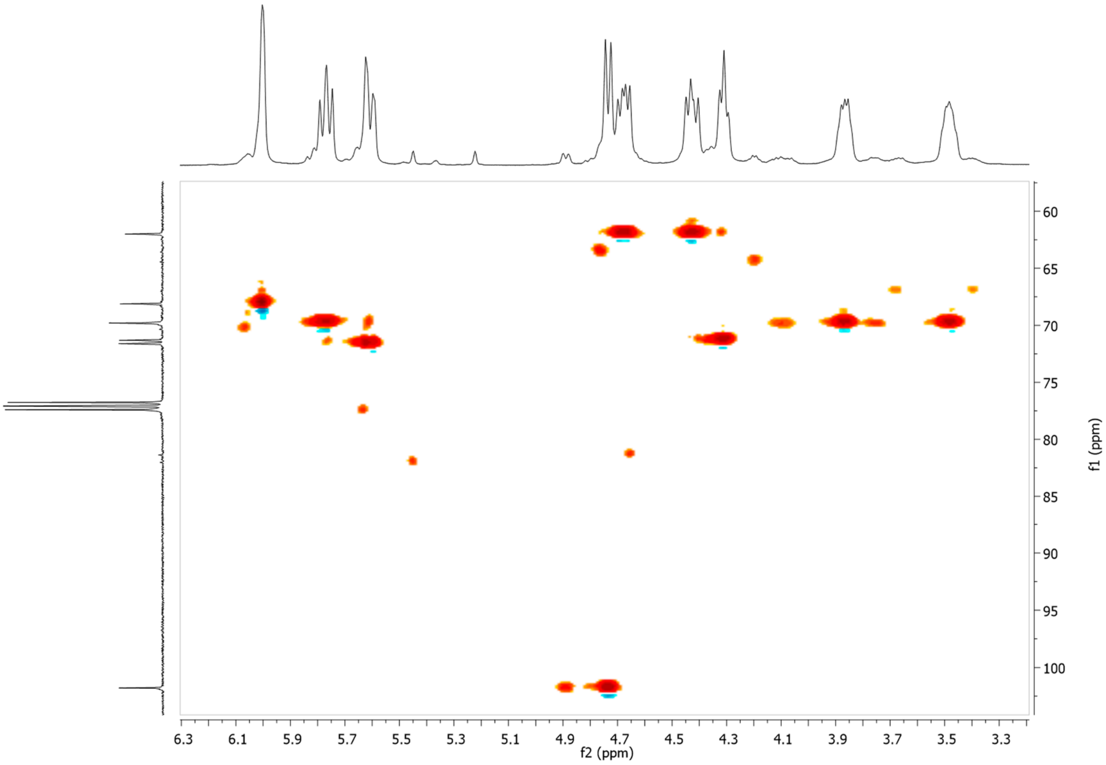

S25. HMQC expansion of bis(1-*O*-ethyl-2,3,4,6-tetra-*O*-benzoyl-β-D-galactopyranosyl)amine, **9**.

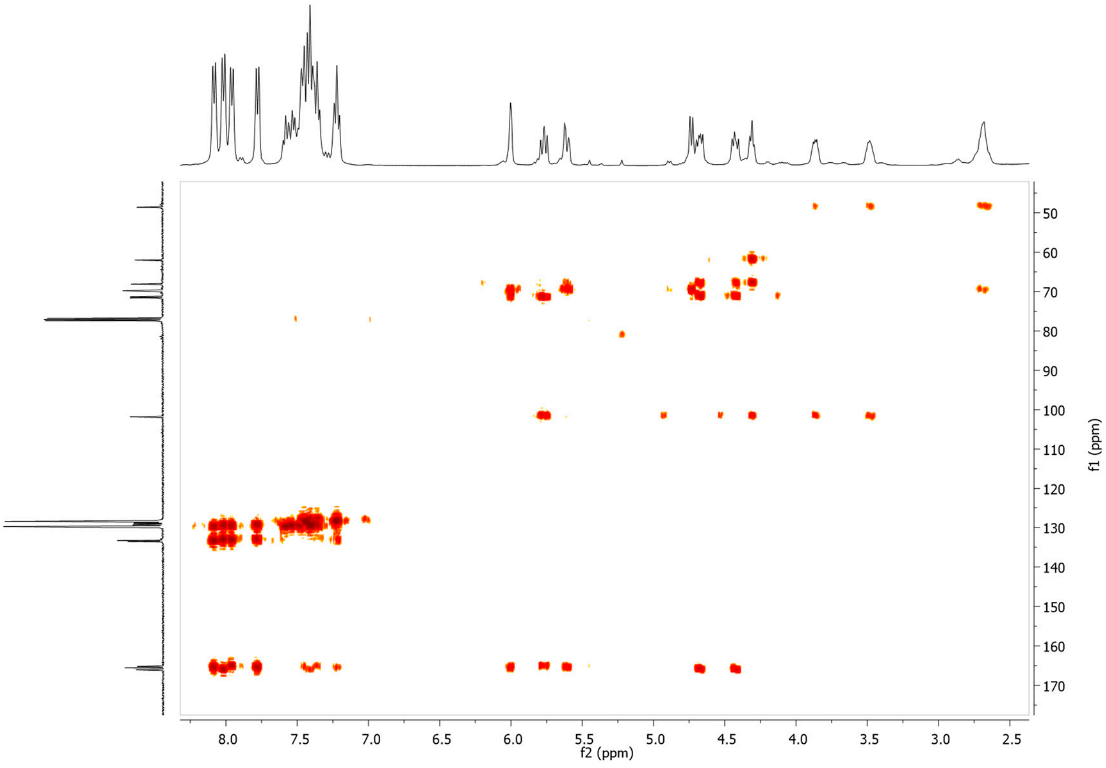

S26. HMBC spectrum ($CDCl_3$) of bis(1-*O*-ethyl-2,3,4,6-tetra-*O*-benzoyl-β-D-galactopyranosyl)amine, **9**.

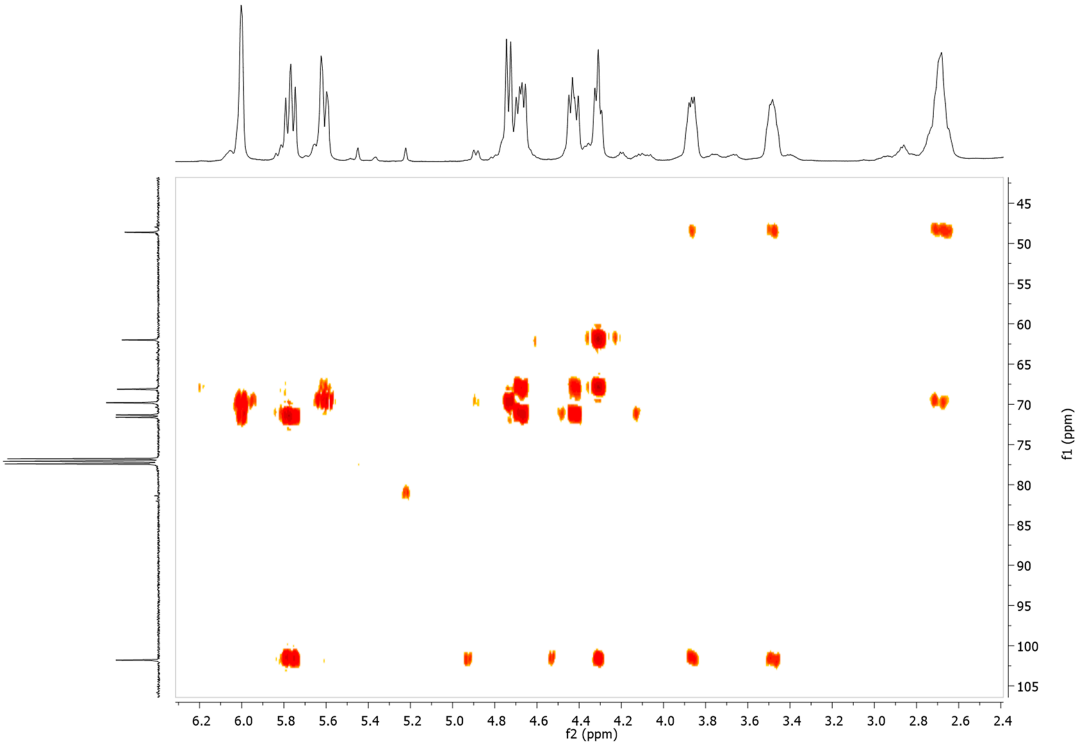

S27. HMBC expansion of bis(1-*O*-ethyl-2,3,4,6-tetra-*O*-benzoyl-β-D-galactopyranosyl)amine, **9**.

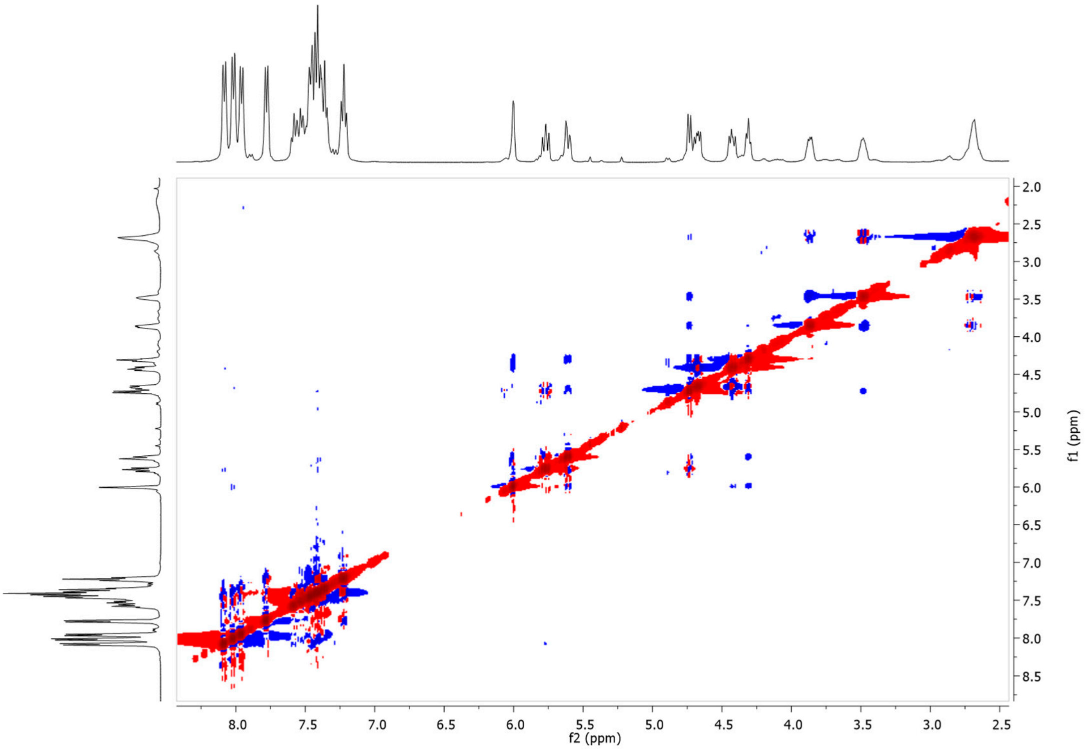

S28. NOESY spectrum ($CDCl_3$) of bis(1-*O*-ethyl-2,3,4,6-tetra-*O*-benzoyl-β-D-galactopyranosyl)amine, **9**.

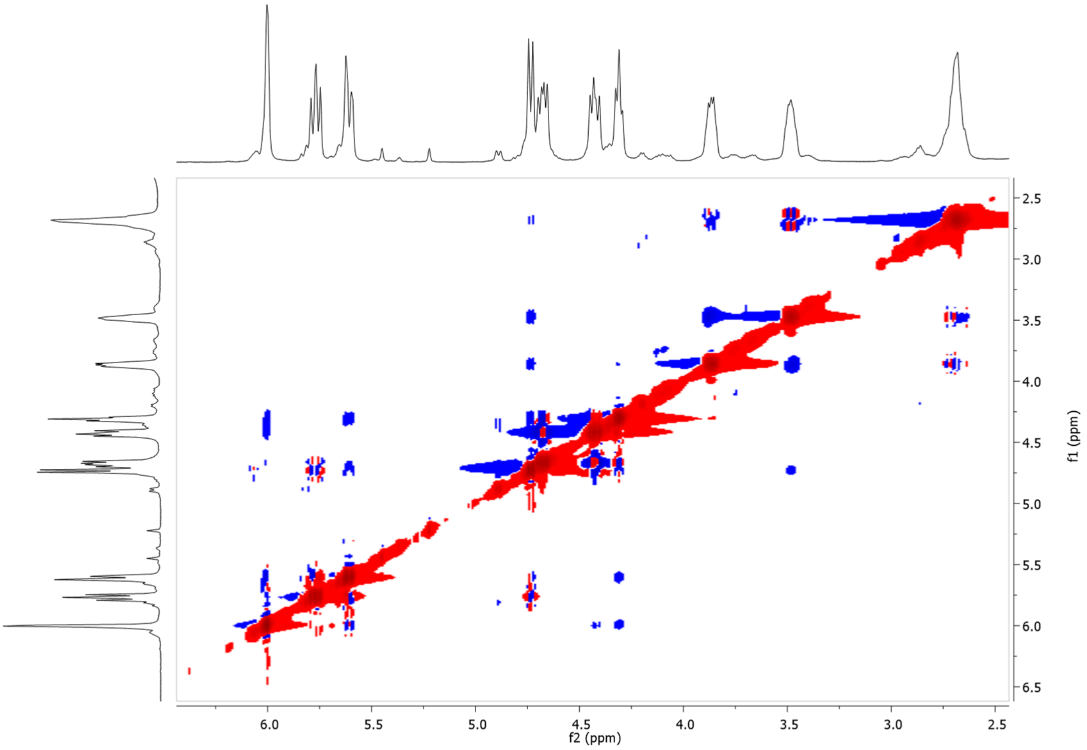

S29. NOESY expansion of bis(1-*O*-ethyl-2,3,4,6-tetra-*O*-benzoyl-β-D-galactopyranosyl)amine, **9**.

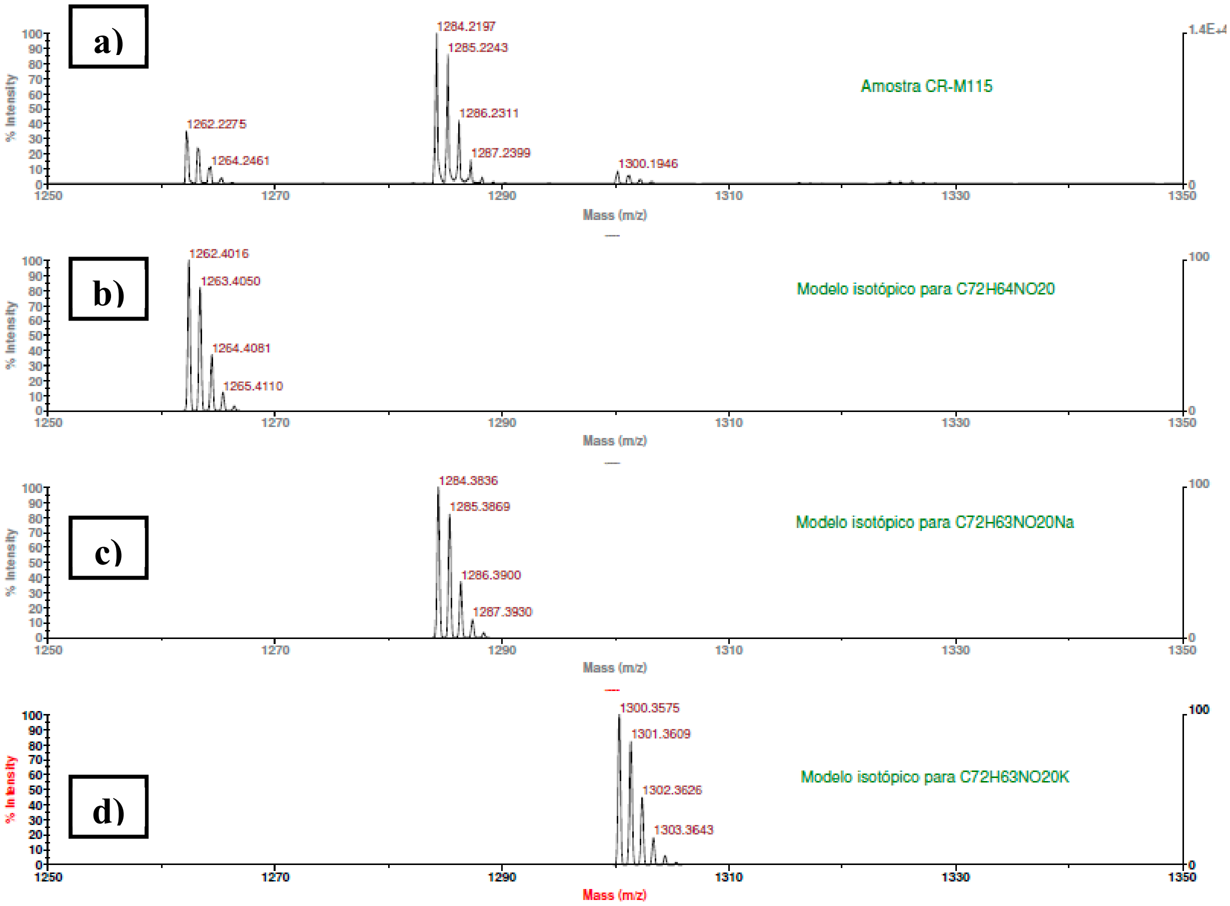

S30. MALDI-TOF mass spectra of a) bis(1-*O*-ethyl-2,3,4,6-tetra-*O*-benzoyl-β-D-galactopyranosyl)amine, **9** sample [$C_{72}H_{64}NO_{20}$ (M^+)], and isotope models for b) $C_{72}H_{64}NO_{20}$ c) $C_{72}H_{64}NO_{20}Na$ and d) $C_{72}H_{64}NO_{20}K$.

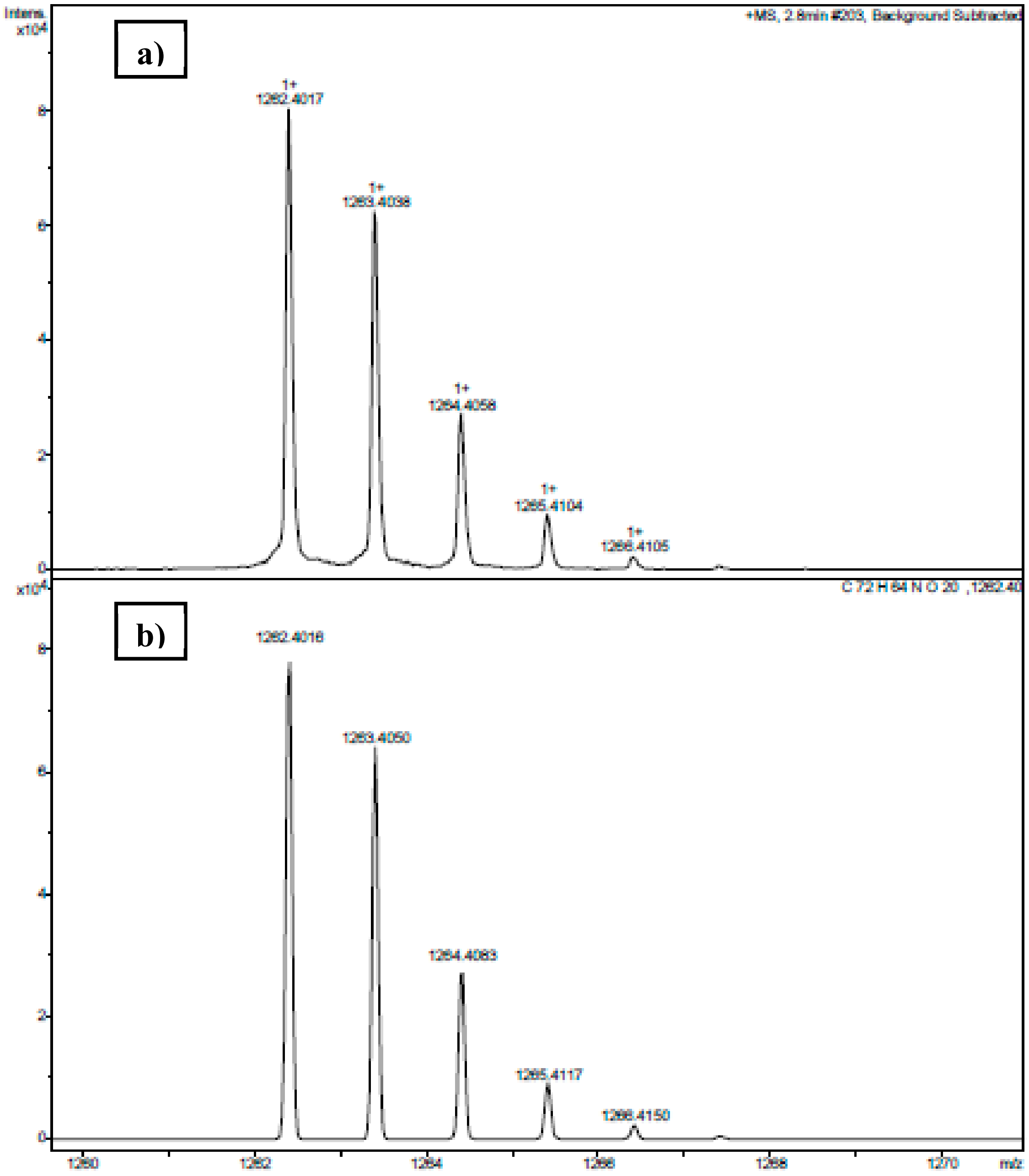

S31. ESI-FIA-TOF mass spectra of a) bis(1-*O*-ethyl-2,3,4,6-tetra-*O*-benzoyl-β-D-galactopyranosyl)amine, **9** **[**$C_{72}H_{64}NO_{20}$ (M^+)], and b) isotope model for $C_{72}H_{64}NO_{20}$ (M^+).

Bis(1-O-ethyl-β-D-galactopyranosyl)amine, 3a

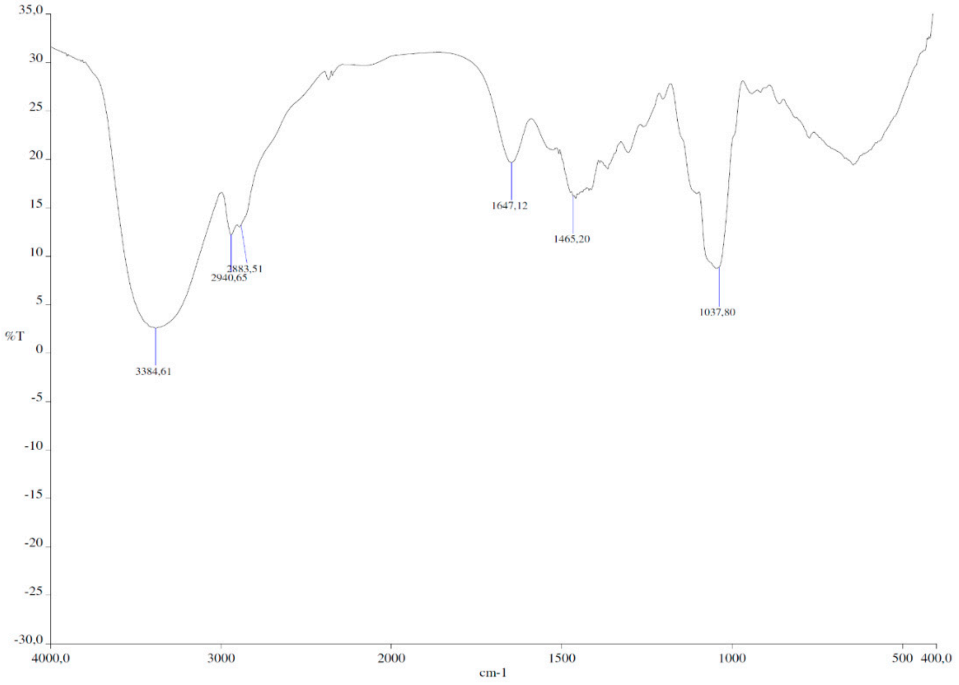

S32. FTIR spectrum of bis(1-*O*-ethyl-β-D-galactopyrano*syl*)amine, **3a**.

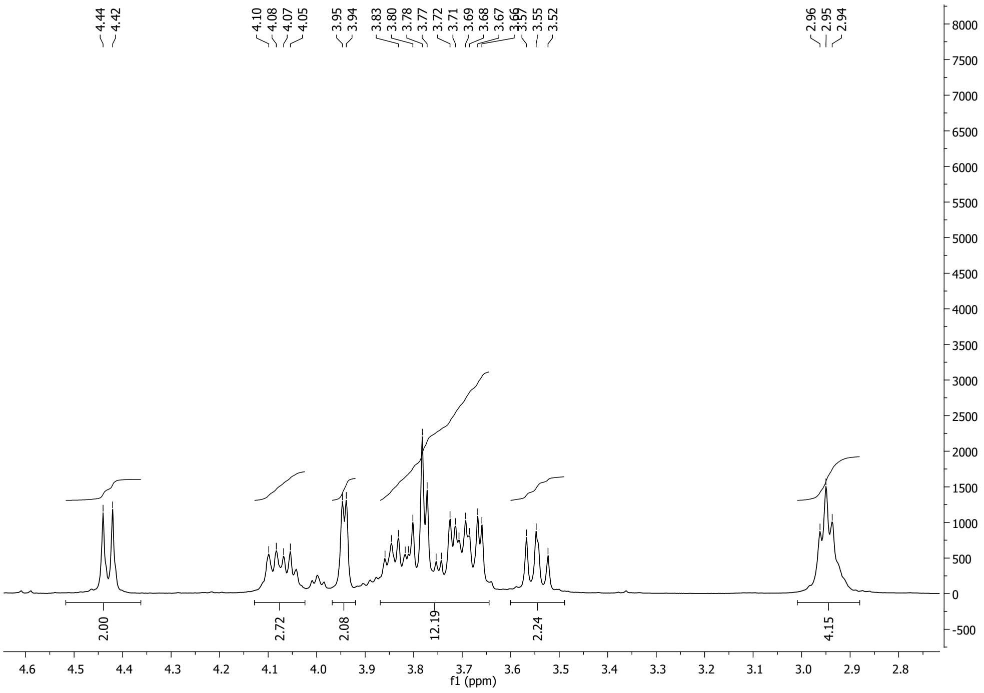

S33. ^{1}H NMR spectrum (D_2O) of bis(1-*O*-ethyl-β-D-galactopyrano*syl*)amine, **3a**.

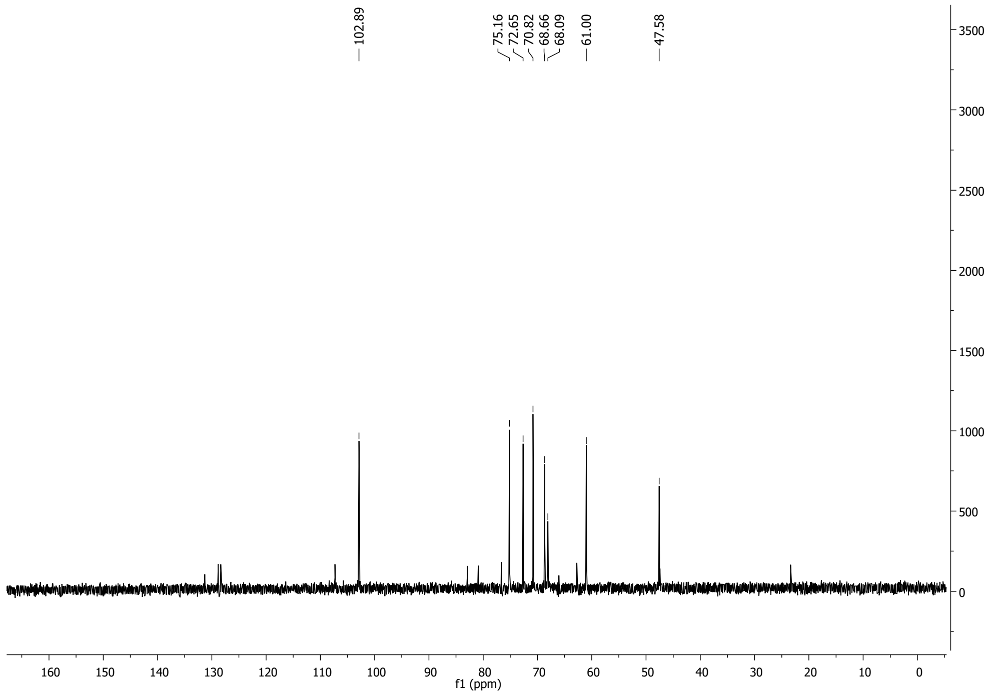

S34. ^{13}C NMR spectrum (D_2O) of bis(1-*O*-ethyl-β-D-galactopyrano*syl*)amine, **3a**.

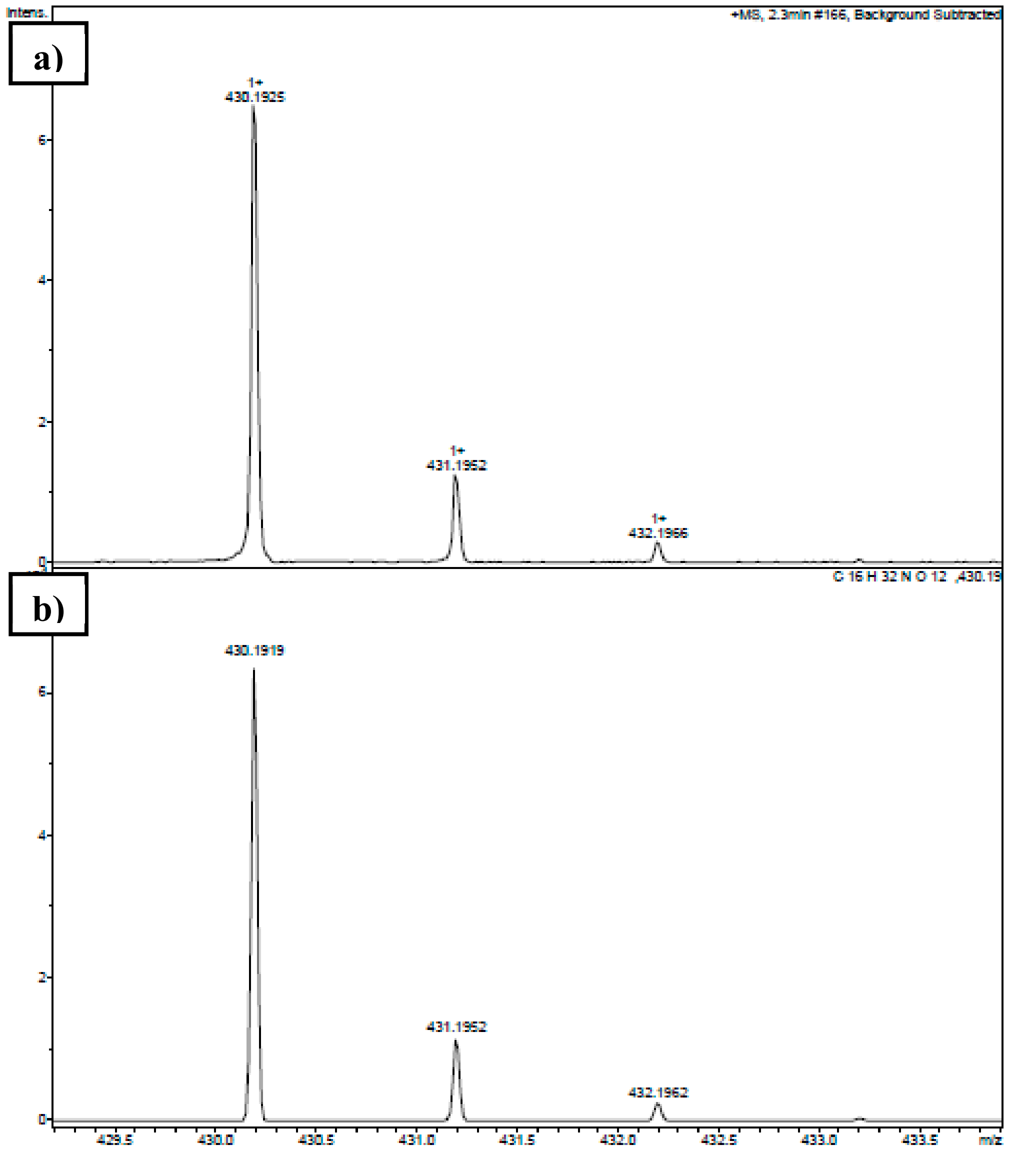

S35. ESI-FIA-TOF mass spectra of a) bis(1-*O*-ethyl-β-D-galactopyrano*syl*)amine, **3a** [$C_{16}H_{32}NO_{12}$ (M^+)], and b) isotope model for $C_{16}H_{32}NO_{12}$ (M^+).

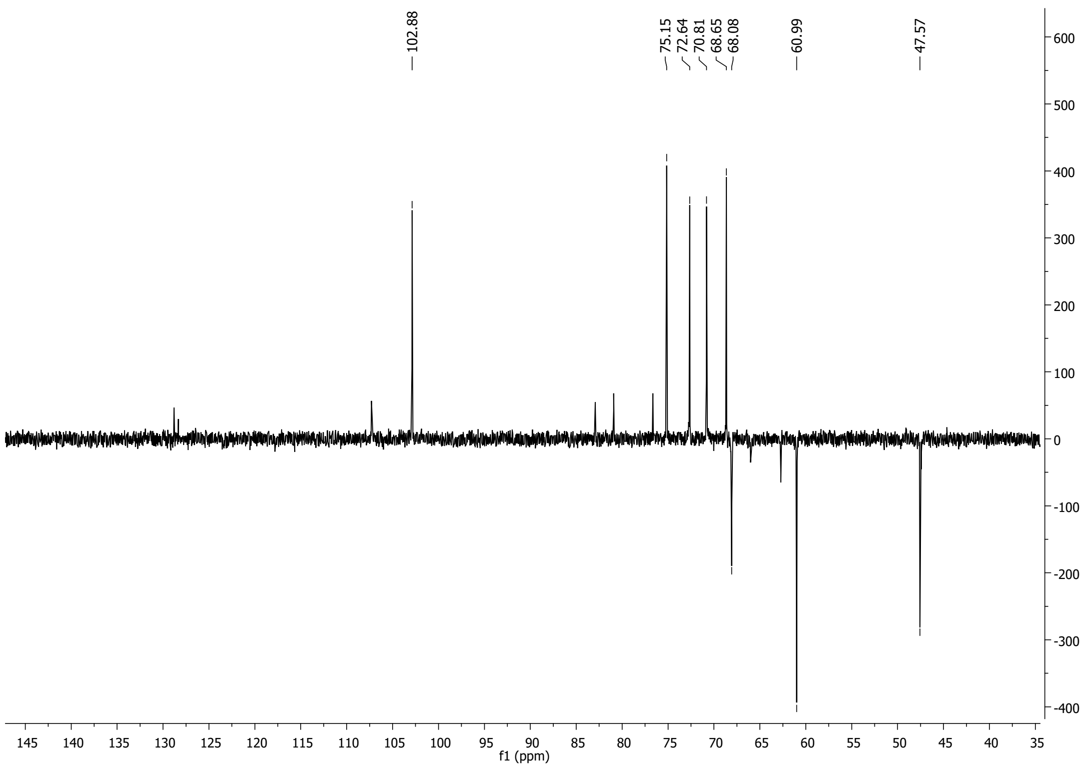

S36. DEPT spectrum (D_2O) of bis(1-*O*-ethyl-β-D-galactopyrano*syl*)amine, **3a**.

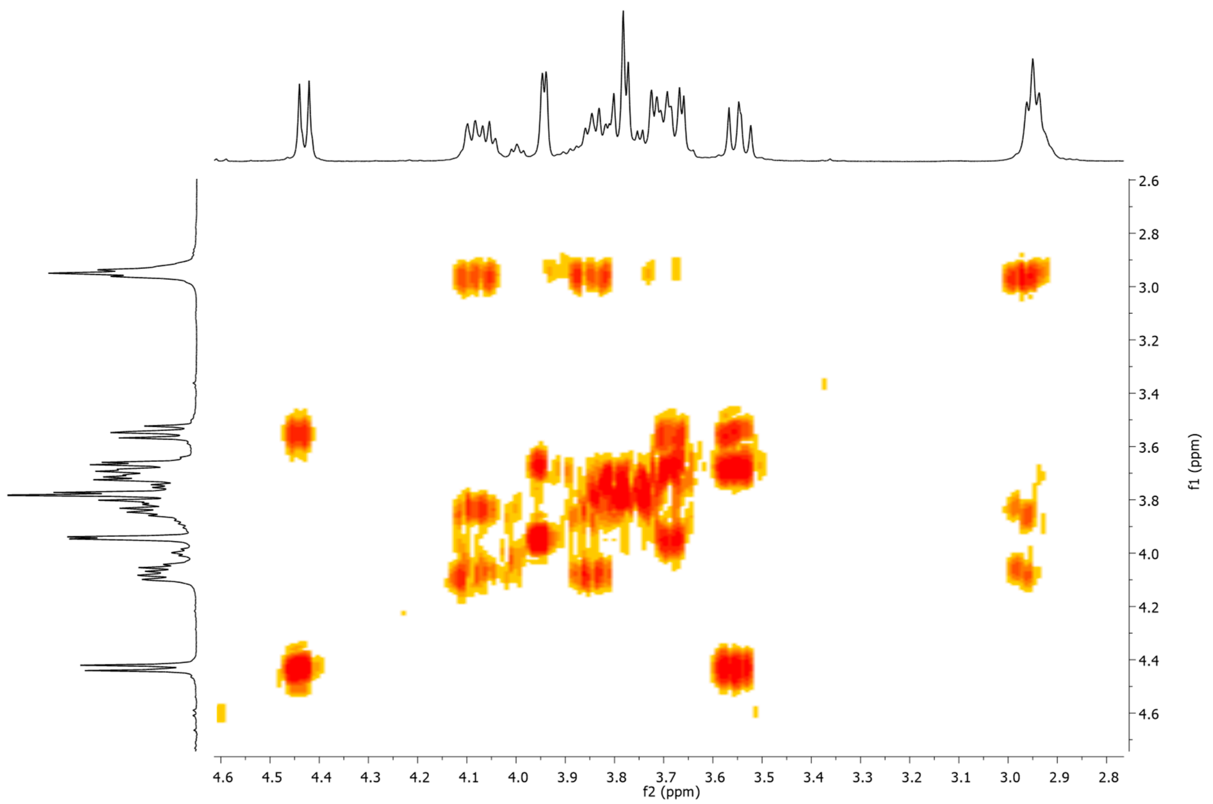

S37. COSY spectrum (D_2O) of bis(1-*O*-ethyl-β-D-galactopyrano*syl*)amine, **3a**.

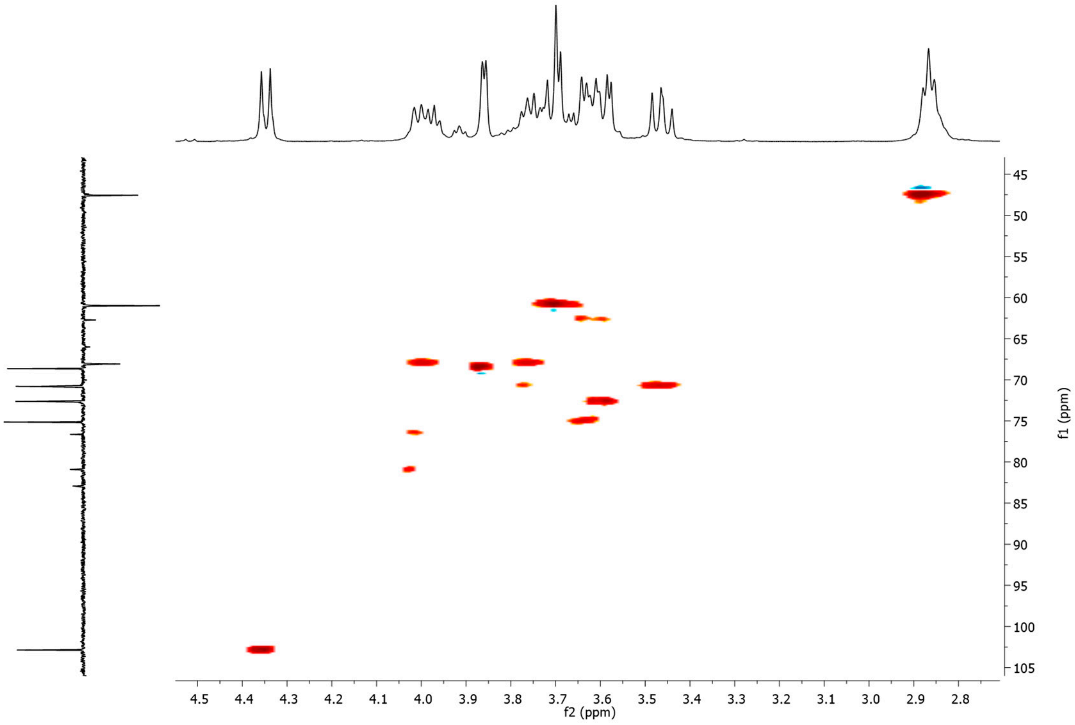

S38. HMQC spectrum (D_2O) of bis(1-*O*-ethyl-β-D-galactopyrano*syl*)amine, **3a**.

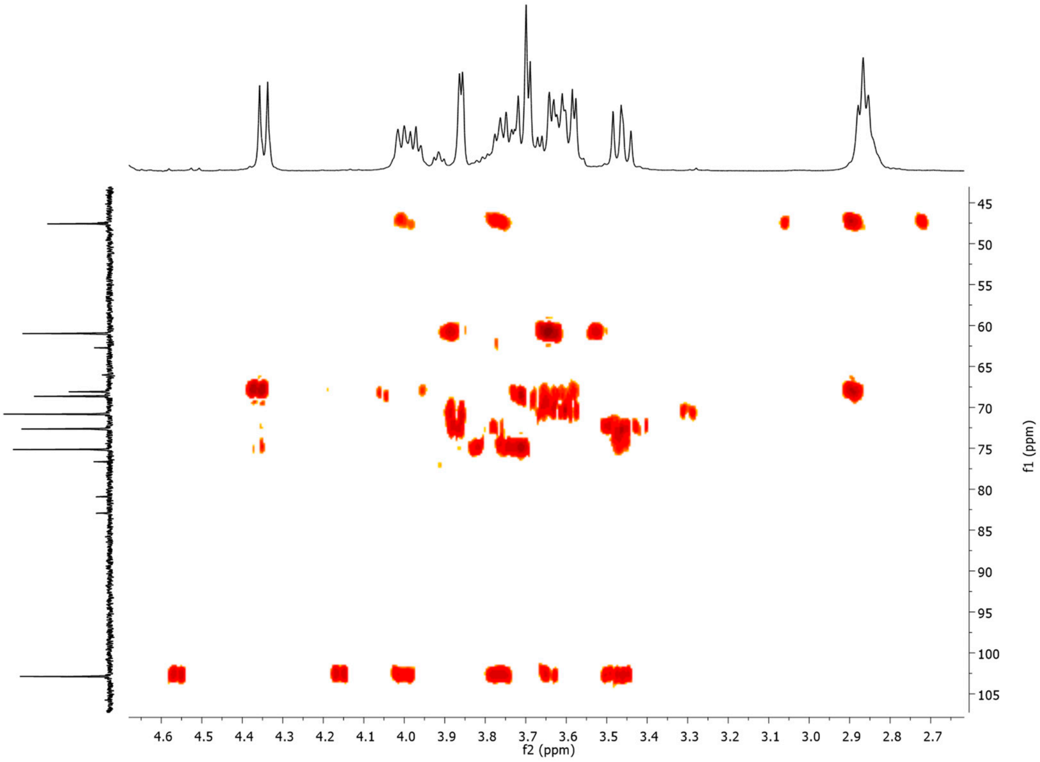

S39. HMBC spectrum (D_2O) of bis(1-*O*-ethyl-β-D-galactopyrano*syl*)amine, **3a**.

Bis(1-O-ethyl-β-D-galactopyranosyl)amine-PLGA conjugate, PLGA-di-GAL, 10

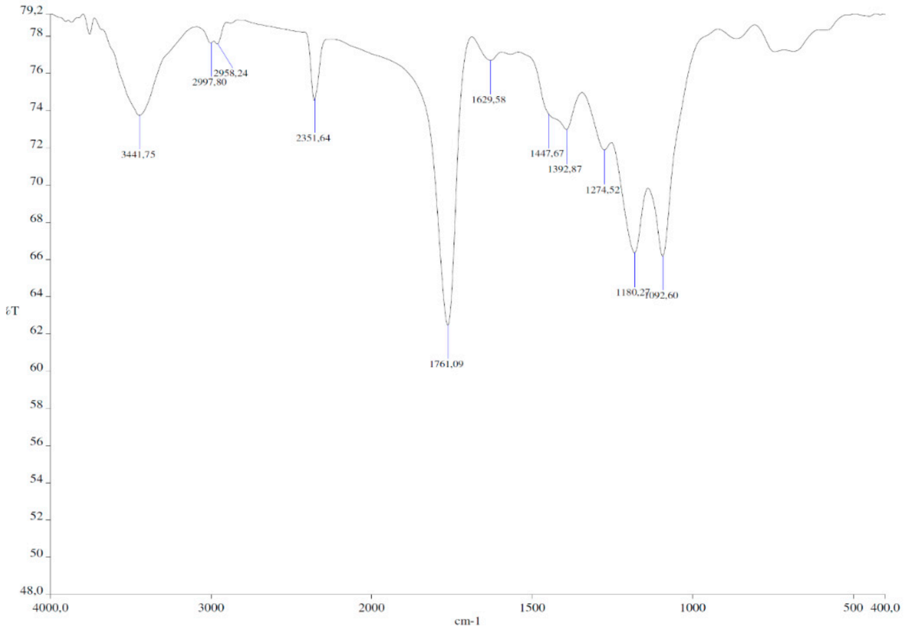

S40 . FTIR spectrum of bis(1-O-ethyl-β-D-galactopyranosyl)amine-PLGA conjugate, PLGA-di-GAL, **10.**

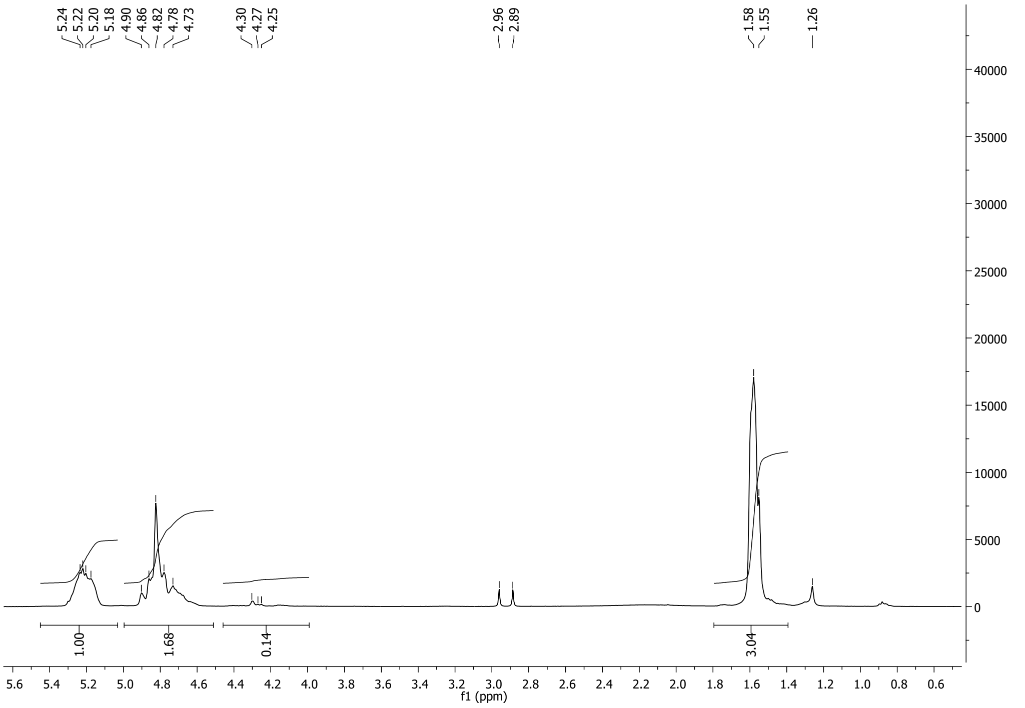

S41. ^{1}H NMR spectrum (D_2O) of bis(1-*O*-ethyl-β-D-galactopyrano*syl*)amine-PLGA conjugate, PLGA-di-GAL, **10**.

4- Characterization of NP

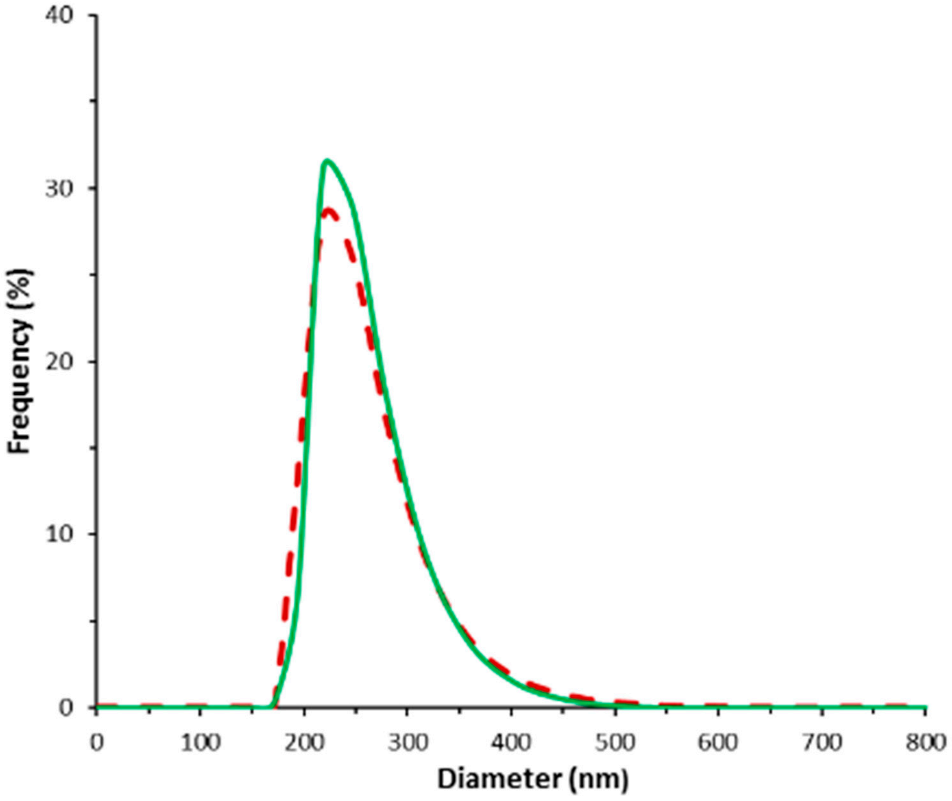

S42. Particles size distribution of PLGA NP (dashed line) and PLGA-di-GAL NP (solid line).

References

20. Shendage, D.; Frohlich, R.; Haufe, G. Highly Efficient Stereoconservative Amidation and Deamidation of α-Amino Acids. *Organic Lett.* **2004**, *6 (21)*, 3675-3678. DOI: 10.1021/ol048771l.

23. Goldschmid, H.R.; Perlin, A.S. Some Factors Affecting The Konigs-Knorr Synthesis of Glycosides. *Canadian J. Chem.* **1961**, *39*, 2025-2034. DOI: 10.1139/v61-272.

24. Kimmel, R.; Kafka, S.; Kosmrlj, J. Selective formation of glycosidic linkages of N-unsubstituted 4-hydroxyquinolin-2-(1H)-ones. *Carbohyd. Res.* **2010**, *345 (6),* 768-779. DOI: 10.1016/j.carres.2010.01.023.

25. Stick, R.V.; Williams, S.J. Carbohydrates: the essential molecules of life. Elsevier: Amsterdam; 2009.

34. Mohapatra, D. K.; Durugkar, K. A. Efficient and selective cleavage of the tert-butoxycarbonyl (Boc) group under basic condition. Arkivoc, 2005, xiv, 20-28. DOI: 10.3998/ark.5550190.0006.e03.35.

35. Yamazoe, A.; Hayashi, K.; Kuboki, A.; Ohira, S.; Nozaki, H. The isolation, structural determination, and total synthesis of terfestatin A, a novel auxin signaling inhibitör from Streptomyces sp. *Tetrahedron Letters*, **2004**, *45*, 8359–8362. DOI: 10.1016/j.tetlet.2004.09.055.

33. Huang, J.; Xu, Z.; Yang, Y. Low-Work-Function Surface Formed by Solution-Processed and Thermally Deposited Nanoscale Layers of Cesium Carbonate. *Advanced Functional Materials*, **2007**, *17*, 1966-1973. DOI: 10.1002/adfm.200700051.
